# Fisher’s Geometric Model as a Tool to Study Speciation

**DOI:** 10.1101/cshperspect.a041442

**Published:** 2024-01-22

**Authors:** Hilde Schneemann, Bianca De Sanctis, John J. Welch

**Affiliations:** 1 Department of Genetics, University of Cambridge, Cambridge CB2 3EH, United Kingdom; 2 Department of Zoology, University of Cambridge, Cambridge CB2 3EJ, United Kingdom

## Abstract

Interactions between alleles and across environments play an important role in the fitness of hybrids and are at the heart of the speciation process. Fitness landscapes capture these interactions and can be used to model hybrid fitness, helping us to interpret empirical observations and clarify verbal models. Here, we review recent progress in understanding hybridization outcomes through Fisher’s geometric model, an intuitive and analytically tractable fitness landscape that captures many fitness patterns observed across taxa. We use case studies to show how the model parameters can be estimated from different types of data and discuss how these estimates can be used to make inferences about the divergence history and genetic architecture. We also highlight some areas where the model’s predictions differ from alternative incompatibility-based models, such as the snowball effect and outlier patterns in genome scans.

## What Are Fitness Landscapes, And How Are They Useful For Studying Speciation?

Most of the progress in speciation research has been driven by empirical work and verbal theories, with mathematical theory playing a relatively minor role (Turelli et al. 2001). Nevertheless, mathematical models have made some important contributions; these include unification—allowing us to compare, contrast, and combine different speciation scenarios (Felsenstein 1981; Turelli et al. 2001; Gavrilets 2004, 2014), and data interpretation—telling us what we can and cannot infer from a particular empirical observation (e.g., Dobzhansky 1937; Barton and Gale 1993; Rundle and Whitlock 2001; Schumer and Brandvain 2016; Baird 2017; Li et al. 2022).

Many questions about speciation concern the fitness of hybrids. Reduced hybrid fitness is, of course, a central component of reproductive isolation, and so we would like to understand the evolution of postzygotic barriers and their persistence or collapse upon secondary contact (Coyne and Orr 2004; Bierne et al. 2011; Bank et al. 2012; Xiong and Mallet 2022). But hybrids sometimes display a fitness advantage, and another set of questions involve hybrid speciation, or the role of introgression in hindering or driving divergence—especially during adaptive radiations (Anderson 1949; Seehausen 2004; Abbott et al. 2013; Marques et al. 2019; Peñalba et al. 2023).

To address the questions above, we need to model hybridization in a way that connects properties of genotypes to their fitness—in other words, we need some kind of fitness landscape model (Dobzhansky 1937; Orr 1995; Gavrilets 2004; Fragata et al. 2019). While the term “fitness landscape” can mean many things, all we mean here is a mapping between genotype and fitness. In other words, if we were simulating evolution and a new genotype appears in the simulation, a fitness landscape model tells us how we should assign its fitness. Fitness landscape models therefore embody assumptions about the fitness effects of alleles, and the fitness interactions between them. This means they can also be used to make predictions about particular classes of genotype (say, genotypes with more or less heterozygosity), and about the evolutionary dynamics of divergence and of hybridization under different conditions.

Many different fitness landscape models have been used in the study of speciation, and a few examples are listed in Table 1. All the landscapes in Table 1 are rugged to some extent, with multiple genotypic fitness peaks. They therefore meet the most basic requirement for the study of speciation genetics—that alleles can be beneficial in one genomic context, and deleterious in another. Moreover, all the landscapes capture the seminal insight of the field—that populations may diverge to a point where their hybrids are very unfit, but without either population passing through a state of low fitness during their divergence (Dobzhansky 1937; Gavrilets 2014).

## Fisher’s Geometric Model As A Fitness Landscape

### How Does Fisher’s Geometric Model Map Genotype to Fitness?

In this article we focus on the last entry in Table 1, namely, Fisher’s geometric model (Fisher 1930; Lande 1980; Turelli 1985; Orr 1998). Under this model, fitness values are assigned to genotypes using a simple and idealized model of optimizing selection acting on *n* continuously varying traits. Each new allele has fixed additive and dominance effects on one or more of the traits, and these effects are summed across loci to obtain the trait values. For example, the value of trait *i*, denoted *z*_*i*_, might be written as

(1) $$z_{i}=z_{\text{P}1,i}+\underset{j\in \;\text{hom}\;}{\sum }2a_{ij}+\underset{j\in \;\text{het}\;}{\sum }\left(a_{ij}+d_{ij}\right)$$

where *z*_P1,*i*_ is the trait value of a reference genotype—chosen here to be a typical individual from one of the parental populations; *a*_*ij*_ and *d*_*ij*_ are the additive and dominance effects of the alternative allele at locus *j*; and the sums are across loci that are either homozygous or heterozygous for the alternative allele. Fitness depends on the deviation of all of the trait values from their optimal values, which are determined by the current environment. For example, we might define relative fitness as

(2) $$w\equiv \exp\left[-{\left(\sum_{i=1}^{n}{\left(z_{i}-o_{i}\right)}^{2}\right)}^{k/2}\right]$$

where *o*_*i*_ is the current optimal value for trait *i*. Equation 2 uses the Euclidean distance of the phenotype from the optimum, and assumes that traits contribute independently to fitness. Different authors have made different assumptions (e.g., Peck et al. 1997; Barton 2001; Martin et al. 2007; Chevin et al. 2014; Fraïsse and Welch 2019), but most of these differences have little qualitative effect on the predictions for hybrids. Far more influential is the parameter *k*, which determines the overall rate at which fitness declines with the distance to the optimum, and which we discuss in the next section below.

Given Equations 1 and 2, and the complete set of the *a*_*ij*_ and *d*_*ij*_, we can assign a fitness value to all possible genotypes. Moreover, from knowledge of the distributions of *a*_*ij*_ and *d*_*ij*_, we can make predictions about the typical fitnesses of different classes of genotype (see below). Note also that the description above encompasses important models of speciation, such as that of Mani and Clarke (1990), even though those authors did not use the term “Fisher’s geometric model.”

### A Phenotypic Model or a Fitness Landscape?

The model described above is often treated primarily as a phenotypic model, and used to study the evolution of quantitative traits (i.e., the evolution of the *z*_*i*_) (e.g., Lande 1980; Turelli 1985; Orr 1998; Simons et al. 2018; Hayward and Sella 2022). This use of the model is related to important work in the study of speciation that also focuses on phenotypes (e.g., Christie and Macnair 1987; Schluter 2000; Jiggins et al. 2001; Coyne and Orr 2004; Lehmann and Diabate 2008; Seehausen et al. 2008; Grant and Grant 2009; Shaw and Mullen 2011).

However, none of that is the focus of the present article. Here, we make no claims for the realism of the phenotypic model, but view it solely as a fitness landscape (Martin et al. 2007) that should be compared to those listed in Table 1. The phenotypic model is simply a way of deriving predictions about genotypic fitnesses, and the underlying population genetic parameters (e.g., the distributions of selection coefficients, dominance coefficients, and epistatic interactions). The model’s most important property is that a given change to the phenotype can have different fitness effects in different genetic backgrounds (i.e., according to other alleles carried), and in different environments (i.e., according to the position of the optimum). This means that alleles from one species can be incompatible with alleles in the heterospecific background, or that hybrid genotypes can outperform parental types in a limited part of the range. This is how the model incorporates the context-dependence that is at the heart of speciation genetics (Bordenstein and Drapeau 2001; Hietpas et al. 2013; Fragata et al. 2019; Brice et al. 2021; Thompson et al. 2022, 2023).

### Why this Fitness Landscape?

Many different fitness landscapes have the context-dependence properties mentioned above, or are easily extended to do so (Bordenstein and Drapeau 2001; Hietpas et al. 2013; Fragata et al. 2019; Brice et al. 2021; Thompson et al. 2022), and it is generally a bad idea to focus too exclusively on one particular modeling approach, especially when the key parameters can seem opaque (see below). Nevertheless, we think that Fisher’s model does combine tractability and flexibility in a way that can be fruitful for speciation research.

Indeed, the model has already proven useful for many questions related to speciation, some of which are listed in Table 2. A notable strength of the model is that it allows us to connect these questions to related questions about within-species evolution. For example, the model has long been used to investigate the properties of mutations fixed by evolving populations (e.g., Orr 1998; Trindade et al. 2012; Blanquart et al. 2014; Matuszewski et al. 2014) and the evolution of genetic architectures (Simons et al. 2018; Yeaman 2022; Laroche and Lenormand 2023). Moreover, many speciation scenarios were first systematically explored with models of optimizing selection on quantitative traits, including speciation via divergent ecological selection (Schluter 2000; Nosil 2012), mutation-order speciation (Mani and Clarke 1990; Johnson and Porter 2000; Barton 2001), system drift in constant environments (Barton 1989; Johnson and Porter 2000; Taylor and Higgs 2000; Fierst and Hansen 2010; Rosas et al. 2010; Schiffman and Ralph 2022), founder effects and bottlenecks (Barton and Charlesworth 1984; Gavrilets and Hastings 1996; Yamaguchi et al. 2022), and speciation involving hybridization (Rieseberg et al. 1999; Barton 2001; Yamaguchi and Otto 2020; Kulmuni et al. 2023). By combining these results with the study of hybridization, we can ask whether particular patterns of hybrid fitness follow predictably from particular histories of divergence (Chevin et al. 2014; Simon et al. 2018; Schneemann et al. 2020; Yamaguchi et al. 2022; De Sanctis et al. 2023; see also below). The flexibility of trait-based models also implies that they can be extended to include other factors that are important to speciation, such as frequency-dependent and disruptive selection (Lande 1976; Dieckmann and Doebeli 1999; Gavrilets 2014), sexual selection, prezygotic barriers and reinforcement (Lande 1981; Iwasa et al. 1991; Liou and Price 1994; Turelli et al. 2001; Gavrilets 2014), and genetic conflicts (Haig and Westoby 1991; Connallon and Clark 2014; Rautiala and Gardner 2023). In each case, this involves extending the fitness function of Equation 2, for example, such that fitness depends on the state of the population.

A final strength, paradoxically, is that Fisher’s geometric model makes very similar predictions to some other fitness landscape models, at least for some kinds of data (e.g., Martin 2014; Fraïsse et al. 2016; Simon et al. 2018; Reddy and Desai 2021; Cotto and Day 2023). This is generally a positive thing since we can have more confidence in predictions that do not rely on the details of any particular model. Nevertheless, it reminds us that the match between data and prediction does not prove that the process modeled was responsible for generating the data and also makes it especially important to focus on the cases where the predictions differ (see below).

## Interpretation And Applications

In the remaining sections of this article, we aim to clarify the key parameters and predictions of Fisher’s geometric model and its relation to other approaches. Throughout, we demonstrate how the model can be used to interpret data, from controlled crosses and reciprocal transplants to introgression lines and genome scans.

### What Do the Model Parameters *n* and *k* Mean?

**Figure 1:**
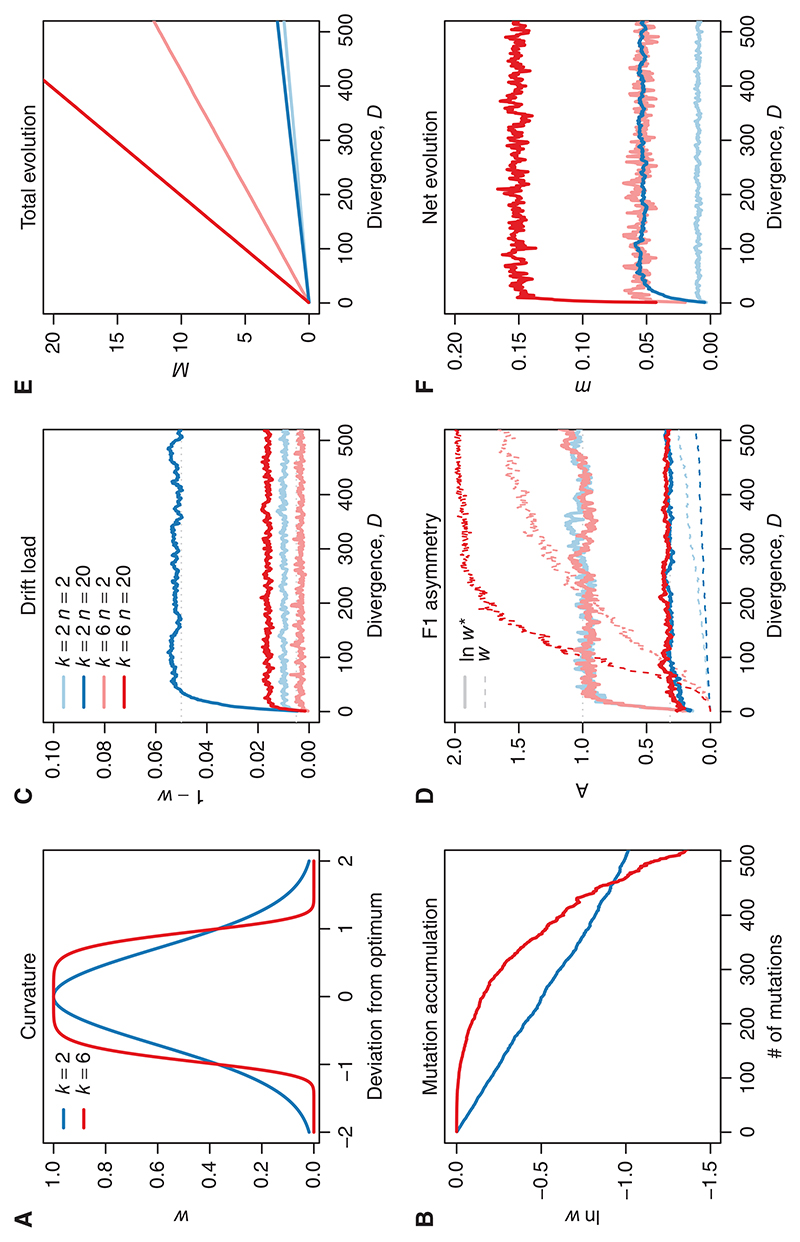
Effects of fitness landscape parameters *k* and *n*. (*A*) The parameter *k* determines the rate with which fitness declines with distance to the optimum. Analytical results are easier to obtain with *k* = 2 (blue lines), but some aspects of the speciation genetic data are more consistent with a higher *k* landscape (e.g., *k* = 6 shown in red). (*B*) *k* also determines typical levels of epistasis, and thereby the rate at which log fitness declines in a mutation accumulation experiment; with *k* = 2, mutations act independently on average so that each new mutation reduces fitness by the same fraction on average; *k* > 2 generates negative epistasis so that each additional mutation reduces fitness by more than the last (this result applies regardless of the mutation model, as long as mutations have no tendency to act in the same phenotypic direction; and in all cases there will be variation in epistasis around these expected values, with lower *n* models showing higher variance; Fraïsse and Welch 2019). (*C*–*F*) Individual-based simulations with low and high values of *k* (blue vs. red lines) and *n* (light vs. dark lines). Lines represent the mean across 100 replicates over divergence (the number of substitutions differentiating the parental lines, denoted *D*). Simulations involved a Wright–Fisher population of *N*_*e*_ = 100 individuals in a constant environment, with freely recombining, simultaneously segregating mutations with an average selection coefficient of $\bar{\text{s}}=0.01$ in an optimal background, and with variable phenotypic dominance. For simulation details, see Supplemental Appendix S1. (*C*) The drift load in single populations increases with *n*, and when *k* = 2, it is close to its predicted value of *n*/(4*N*_*e*_) (Lande 1976; Barton 2017) as indicated by the gray dotted lines. These costs of high *n* are maximized when *k* = 2, because negative epistasis (*k* > 2) increases the efficiency of selection (Kondrashov 1984). (*D*) The fitness asymmetry between the reciprocal F1 of two divergent populations varies with *k* and *n* (thin dashed lines), while asymmetry in the transformed fitness (lnw^*^; Equation 3) depends only on *n* (thick solid lines), and matches the predicted value of $\sqrt{2/n}$ (which applies only in the presence of variable phenotypic dominance; see Schneemann et al. 2022) as indicated by the gray dotted lines. Asymmetry is defined via the function $$A(x)\equiv \left|x_{1}-x_{2}\right|/\left(\frac{1}{2}\left(x_{1}+x_{2}\right)\right)$$ where *x*_1_ and *x*_2_ denote the values for the two cross directions. Reciprocal F1 differed by a random portion of ~10% of the genome being uniparentally expressed. (*E,F*) The total amount (*M*) and net effect (*m*) of evolutionary change as described in the main text and De Sanctis et al. (2023). (*E*) *M* increases linearly with divergence at a rate dependent on the effect size of substitutions, which increases with *n* and *k*. (*F*) Like the drift load, *m* remains constant, at a level that increases with *n* and *k*.

In this section, we discuss the two parameters that appear in Equation 2: *k*, which determines how quickly fitness declines with distance from the optimum; and *n*, the number of phenotypic traits under selection. Some of the effects of these parameters are shown in Figure 1.

The basic role of the parameter *k* is evident from the fitness functions shown in Figure 1A. When *k* = 2 (blue curve), fitness declines gradually with the squared distance to the optimum (Lande 1976); but with larger *k* values (red curve), the fitness function becomes more “table-like” (Fraïsse et al. 2016), with a plateau of high fitness values, surrounded by a precipitous drop. In this way, *k* determines typical levels of fitness epistasis between randomly orientated mutations (Peck et al. 1997; Martin and Lenormand 2006; Tenaillon et al. 2007; Roze and Blanckaert 2014; Fraïsse and Welch 2019). With *k* = 2, deleterious mutations act indespendently on average (Martin et al. 2007), so that log fitness declines linearly with the number of mutations carried (Fig. 1B, blue lines; West et al. 1998); by contrast, with *k* > 2, epistasis between deleterious mutations is negative on average, with each new mutation having a stronger effect than the last (Fig. 1B, red lines; West et al. 1998); very large *k* acts like truncation selection—an extreme form of negative epistasis (Kondrashov 1984).

Similar considerations apply to epistasis between heterospecific alleles in hybrids (West et al. 1998). When *k* = 2, epistatic effects tend to be relatively weak (Barton 2001; Chevin et al. 2014), and apply only to pairs of alleles (Martin et al. 2007). By contrast, with *k* > 2, epistatic effects can be strong and complex, so that the model can generate severe negative interactions between small genomic regions, sometimes involving three or more loci (Fraïsse et al. 2016). With very high *k*, the model resembles a holey landscape (Table 1; Gavrilets 1997, 2004), in which all genotypes are either fully fit or fully sterile/inviable (Manna et al. 2011; Fraïsse et al. 2016). Such landscapes generate the “threshold effect,” discussed by Gavrilets (2004, 2014), where reproductive isolation appears suddenly at a given level of divergence.

The role of *k* presents a problem for modelers because setting *k* = 2 makes it much easier to derive analytical predictions. However, some aspects of the speciation genetic data point clearly to a higher *k* landscape. For example, strong complex epistasis is commonly observed in experimental introgressions (Dobzhansky 1937; Barton 2001; Coyne and Orr 2004; Fraïsse et al. 2014, 2016). There are a number of strategies to overcome this problem. First, we can often use the predictions from a *k* = 2 model to investigate fitness rank order (i.e., which hybrids are fitter than others), since these predictions do not depend on *k*. Second, we can use predictions with *k* = 2 as an approximation when fitness differences are small (Barton 2001; Martin 2014), an approach equivalent to approximating curves (such as the red line in Fig. 1B) with straight lines. Last, with enough data, we can sometimes estimate *k* and then transform the fitness data accordingly (Lynch and Walsh 1998, Chap. 11). We will refer to these transformed fitness values as lnw^*^, and note that they are equivalent to the negative squared distance from the optimum:

(3) $$\begin{array}{c} \ln w^{*}\equiv -{\left(-\ln w\right)}^{2/k}\\ =-\sum_{i=1}^{n}{\left(z_{i}-o_{i}\right)}^{2} \end{array}$$

where relative fitness *w* may be estimated directly from a relative fitness proxy such as survival rate, or calculated from absolute fitness values (e.g., offspring number) as *w* = *W*/*W*_0_ where *W*_0_ is the maximum absolute fitness. After transformation, we can use the analytical predictions from the quadratic model by fitting the model to the lnw^*^ values, rather than to fitness directly (see also below).

Now let us consider the second parameter, *n*. This parameter can be a barrier to understanding Fisher’s model as a fitness landscape, because “the number of traits” only makes sense in the context of the idealized phenotypic model. However, in the current context, it can be useful to think of *n* as reflecting a property of the distribution of fitness effects. In particular, $\sqrt{n}$ affects the probability that a randomly oriented change will act in the direction of the optimum, and therefore its chance of being beneficial (Fisher 1930).

For example, when *n* is large, new random mutations are less likely to be beneficial, all else being equal (Fisher 1930). This explains why large *n* populations find it harder to stay close to the optimum, resulting in a higher drift load and reduced ability to adapt to changing conditions (Lande 1980; Hartl and Taubes 1996; Orr 2000; Welch and Waxman 2003; Martin and Lenormand 2006; Tenaillon et al. 2007; Lourenço et al. 2011; Barton 2017; although see Roze and Blanckaert 2014). This “cost of complexity” is shown in Figure 1C, and when *k* = 2, it can be predicted under quite general conditions (Barton 2017; see gray dotted lines in Fig. 1C).

Again, the same logic applies to new combinations of alleles, which first appear in hybrids. Such combinations are less likely to be fortuitously beneficial when *n* is large. This explains why high *n* populations are less likely to generate hybrids that are pre-adapted to novel environmental conditions (Yamaguchi and Otto 2020; Schneemann et al. 2022; Kulmuni et al. 2023). For the same reason, *n* determines the extent of fitness asymmetry between reciprocal F1 hybrids (male–female vs. female–male cross directions), which is aphenomenon known as Darwin’s corollary (Turelli and Moyle 2007). When there is any form of uniparental inheritance (e.g., mitochondria or sex chromosomes), the reciprocal F1 will express different novel combinations of alleles, and when *n* is small, it becomes more likely that one of these combinations will increase fitness, while the other (pointing in the opposite phenotypic direction) will decrease fitness, making one cross direction much fitter than the other (Fraïsse et al. 2016; Schneemann et al. 2022). As shown in Figure 1D (thin dashed lines), the degree of asymmetry in fitness varies over time (with low asymmetry at very low and possibly at very high divergences as well—the latter not shown here; Turelli and Moyle 2007), but asymmetry never reaches very high levels unless *k* > 2 (Fraïsse et al. 2016). However, after transforming the fitness values using Equation 3, we find that asymmetry in the lnw^*^ values (thick solid lines in Fig. 1D) is now a constant, which depends solely on *n*; and we have for the expected asymmetry $E(A)\;\approx \;\sqrt{2/n}$ (Schneemann et al. 2022).

To illustrate the points above, and show how the model can be applied, let us consider data compiled by Bolnick et al. (2008). These are fitness data from interspecific F1 hybrids of sunfishes (family Centrarchidae) with a range of divergence times between the parents (Near et al. 2005). The proxy for fitness was the hatch rate of F1 hybrid embryos relative to the within-species control cross (see Supplemental Appendix S2 and Bolnick and Near [2005] for full details).

**Figure 2:**
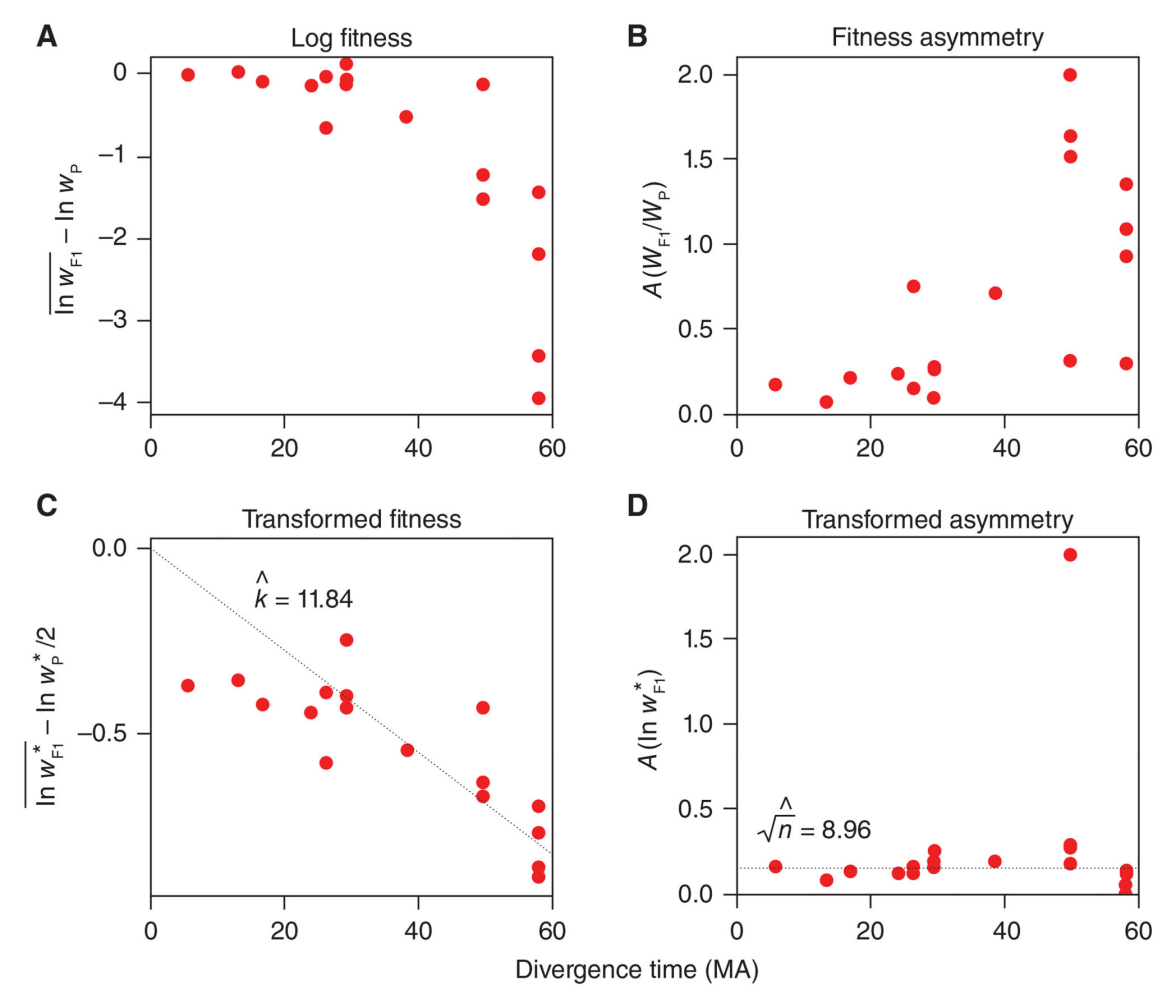
Estimation of *k* and *n* from sunfish data of Bolnick et al. (2008). Hatch rate data from reciprocal F1 crosses (male–female vs. female–male) between different species of sunfish (Centrarchidae), each normalized with the hatch rate of a with in-species control cross (Bolnick and Near 2005; Near et al. 2005; Bolnick et al. 2008). (*A*) F1 log fitness declines rapidly with parental divergence. (*B*) The asymmetry in normalized fitness values increases with divergence. (*C*) shows the fitness values transformed to lnw^*^ values, which decrease approximately linearly with divergence time (see Supplemental Appendix S2 for full details). This transformation involves estimating the curvature parameter, *k*, whose best-fit value is found to be $\hat{k}\approx 11.84$. (*D*) For the lnw^*^ values, the asymmetry between reciprocal F1 is roughly constant with divergence (with a single outlier caused by zero-valued fitness for one cross direction). The constant level of asymmetry gives us a rough estimate of dimensionality $\hat{\sqrt{n}}=8.96$ (Schneemann et al. 2022). (*B,D*) Asymmetry is defined via the function $$A(x)\equiv \left|x_{1}-x_{2}\right|/\left(\frac{1}{2}\left(x_{1}+x_{2}\right)\right)$$ where *x*_1_ and *x*_2_ denote the values for the two cross directions as in Figure 1.

Figure 2A plots the mean log fitness of F1 hybrids (averaged across cross directions) and shows that it declines rapidly with the evolutionary distance between the parental species. Figure 2B plots the asymmetry in F1 fitness values, between the two cross directions, showing that it increases with distance, albeit with a large variance. To understand these data further, let us note that both the faster-than linear curve in Figure 2A, and the strong asymmetry in Figure 2B, are consistent with a high *k* fitness landscape. As explained in detail in the Supplemental Appendix S2, we can use the model to estimate the value of *k* that best characterizes the data in Figure 2A, which allows us to transform the fitness data into lnw^*^ values. As shown in Figure 2C, this transformed F1 log fitness lnw^*^ now declines linearly with evolutionary distance. Moreover, the asymmetry in lnw^*^ shown in Figure 2D takes a much more constrained set of values (except for a clear outlier, caused by a cross with zero-valued hatch rate). As predicted (Fig. 1D; Schneemann et al. 2022), this asymmetry now shows no strong trend with evolutionary distance, which makes it meaningful to estimate the value of *n* that best characterizes the sunfish hatch rate data. Using the crude estimator $\sqrt{n}=\sqrt{2}/\bar{A}$ (Schneemann et al. 2022), we find that $\sqrt{n}\approx 8.96$ and estimates remain comparable if we restrict ourselves to the species that are most closely related (distance < 20MA:$\sqrt{n}\approx 10.98$); of intermediate relatedness (20MA < distance < 40MA:$\sqrt{n}\approx 8.06$); or the most distantly related species (distance > 40 MA:$\sqrt{n}\approx 9.27$).

Taken together, results in this section suggest an analogy between the parameters of Fisher’s model, *k* and *n*, and the well-known population genetic parameter *N*_*e*_ (the effective population size). *N*_*e*_ is a phenomenological parameter, which cannot always be interpreted in a simple way (e.g., as a head count of breeding individuals). And in some cases, different values of *N*_*e*_ will apply to different kinds of data from the same population (Crow and Kimura 1970, Chap. 7). This has sometimes caused confusion, including in the speciation literature (e.g., Barton and Charlesworth 1984). However, the difficulties with interpreting *N*_*e*_ should not cause us to overlook its usefulness. In practice, different measures of *N*_*e*_ will often be quite similar to one another (Barton and Charlesworth 1984), and this helps us to understand how apparently different quantities—such as the depth of genealogies and the efficacy of selection—are, in fact, closely related. This section suggests that the parameters of Fisher’s geometric model, *k* and *n*, may have some of the same weaknesses and strengths (see also Lourenço et al. 2011; Fraïsse et al. 2016).

### How Does the Mode of Divergence Affect Hybrid Fitness?

In this section, we shift focus from *n* and *k*, to consider the properties of allelic effects that differentiate the parental lines, and especially the *a*_*ij*_ that appear in Equation 1. We will show how different modes of divergence lead to systematic differences in the *a*_*ij*_, which lead in turn to differences in the fitness of hybrids. We will focus on predicting the fitness of arbitrary hybrids, as characterized by their genomic composition. For diploid hybrids between two parental lines, P1 and P2, this composition can be captured by the hybrid index, *h*, and interclass heterozygosity, *p*, where *h* is defined as the proportion of the genome with P2 ancestry, and *p* as the proportion of sites with ancestry from both P1 and P2. *h* and *p* can either be measured directly from genomic data (Falush et al. 2003; Fitzpatrick 2012) (without the need to phase the data, or infer recombination breakpoints [Baird et al. 2023]), or their expected values can be inferred from a controlled crossing design, such that, for example, the F2 will have

$$E(h)=E(p)=\frac{1}{2}$$

following the same approach as the quantitative genetic analysis of line crosses (Hill 1982; Lynch 1991; Rundle and Whitlock 2001; Fitzpatrick 2008; Fierst and Hansen 2010).

Now, if we assume the simplest additive phenotypic model (i.e., ignoring dominance effects in Equation 1), then the expected log fitness of the hybrid in a given environment is found to be

(4) $$E(\text{ln}w_{\text{H}}^{*})=\overset{“\text{extrinsic}”}{\overset{︷}{(1-h)\ln w_{\text{P}1}^{*}+h\ln w_{\text{P}2}^{*}}}+\overset{“\text{intrinsic}”}{\overset{︷}{\underset{\text{admixture}}{\underset{︸}{4h(1-h)\times (m-M)}}+\underset{\text{heterozygosity}}{\underset{︸}{p\times M}}}}$$

for any distribution of the allelic effects (for derivation of this result, see De Sanctis et al. 2023; see also Slatkin and Lande 1994; Fierst and Hansen 2010; Chevin et al. 2014; Simon et al. 2018; Schneemann et al. 2020 for comparable results).

The prediction in Equation 4 divides naturally into two terms (Chevin et al. 2014). The first term can be thought of as the “extrinsic” component of hybrid fitness (i.e., the term that is predicted to vary with the current environmental conditions as captured by the position of the optimum). This term is simply the ancestry-weighted sum of the parental fitnesses in the current environment. It will be particularly important when the parents are locally adapted to different environments, or when there is a major asymmetry in their fitness (e.g., when one parent is highly inbred). If hybrids are scored in multiple environments, then only this “extrinsic” part of Equation 4 would change, and there resulting *G* × *E* term would depend solely on the parental fitnesses in these environments (see Table 3; Schneemann et al. 2020), and so could be estimated from “home and away” reciprocal transplants (Rundle and Whitlock 2001; Blanquart et al. 2013).

The second term of Equation 4 does not depend on the position of the optimum, and in that sense, captures the “intrinsic” effects of hybridization. It further divides into the effects of admixture (the term in 4*h*(1 − *h*)), which is the result of segregation variance (Slatkin and Lande 1994) and the effects of heterozygosity (the term in *p*). These two terms depend on two quantities, *m* and *M*, which relate in a simple way to the composite effects of Hill (1982) (see Table 3), and which capture properties of the genetic differences between the parental lineages (the *a*_*ij*_ in Equation 1).

We can think of *M* as the “total amount of evolutionary change” between the parental populations (De Sanctis et al. 2023). Hence, *M* will grow if more genomic differences accumulate (i.e., more and larger differences in allele frequencies between the parental populations), and if these differences have larger effects. This explains how the quantity increases with divergence at a rate that varies with the parameters *n* and *k*, as shown in Figure 1E. In a simple special case with P1 close to the optimum, negligible within-population polymorphism, and *k* = 2, *M* is equal to the sum of the selection coefficients of the substitutions that differentiate the parental lines (top row of Table 3 from Chevin et al. 2014; see also De Sanctis et al. 2023 for a more general definition). *M* will be larger, therefore, if *D* (the number of genomic differences) or $\bar{\text{s}}$ (their mean selective effect) is larger.

By contrast, *m* captures the “net effect of the evolutionary change” between the parental populations. As such, when populations are tracking the same optimum, *m* will depend on how well they have done so. This is shown in Figure 1F, which shows that *m* varies with *n* and *k*, in a way that mirrors their effects on drift load (Fig. 1C). *m* will be especially large when populations are well adapted to different optima (i.e., environments to which a single genotype cannot be well adapted). In general, *m* captures the fitness difference between the populations, under conditions to which one of them is optimally adapted (Chevin et al. 2014; De Sanctis et al. 2023). For example, in our special case where P1 is optimal, the log fitness of P2 is given by $\ln w_{\text{P}2}^{*}=-4m$ (see second row in Table 3).

The way *M* and *m* are defined means that they are expected to have the same value whenever parental lines diverge via the accumulation of randomly orientated mutations, without any selective sieve. As a result, the difference between them, *m* − *M*, contains some information about the form of selection that *has* acted during the process of evolutionary divergence (Chevin et al. 2014; Schneemann et al. 2020; De Sanctis et al. 2023). Moreover, from Table 3, *m* − *M* also quantifies the fitness interactions between the heterospecific alleles. In our special case, for example, *m* − *M* is equal to the sum of their pairwise epistatic fitness effects (Chevin et al. 2014; De Sanctis et al. 2023; see third row in Table 3). This explains why, in Equation 4, *m* − *M* determines the effects of admixture, and therefore the relative fitness of different classes of hybrid.

**Figure 3:**
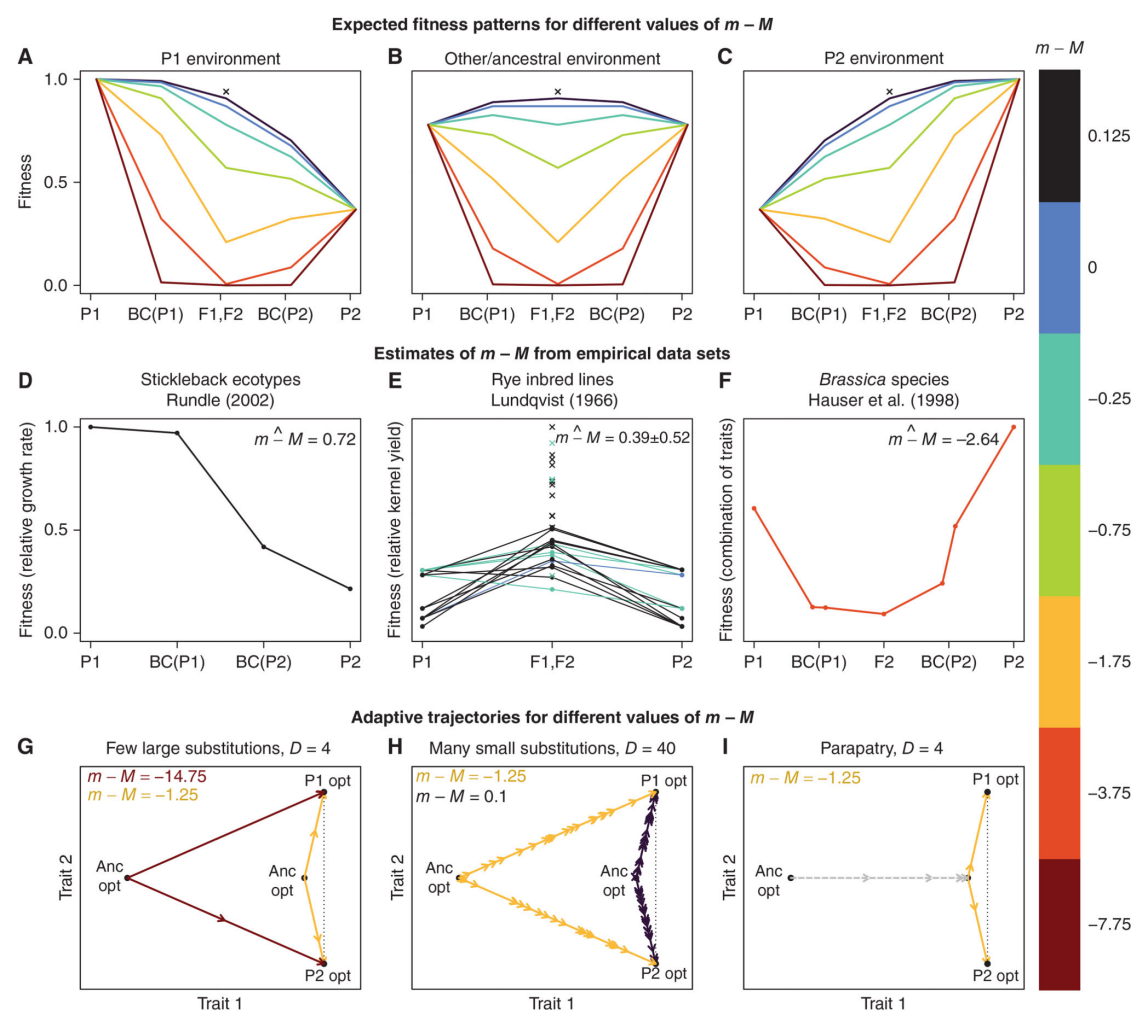
Patterns of hybrid fitness reflect the mode of divergence and epistasis as captured by *m* − *M*. (*A*–*C*) Model predictions for the fitness of parental types (P1: *h* = *p*_12_ = 0; P2: *h* = 1, *p*_12_ = 0), the reciprocal backcrosses (BC(P1): *h* = 1/4, *p*_12_ = 1/2; BC(P2): *h* = 3/4, *p*_12_ = 1/2), the initial F1 hybrid (*h* = 1/2, *p*_12_ = 1; black cross; whose fitness does not depend on *m* − *M*) and the second-generation hybrid (F2: *h* = *p*_12_ = 1/2) in an environment where P1 is well-adapted (*A*), neither parent is well-adapted (*B*), or P2 is well-adapted (*C*), for different values of *m* − *M* as indicated by the color scale on the *right*. When *m* − *M* is positive (black lines), fitness declines rapidly with increasing ancestry from the maladapted parent, indicative of ecological isolation. This pattern becomes weaker as *m* − *M* decreases (blue to redlines), until eventually for very large and negative *m* − *M* (brown lines) we observe a pattern of intrinsic isolation where hybrids between fit parents do poorly across environments. (*D*–*F*) Results for hybrids between stickleback ecotypes (Rundle 2002), rye inbred lines (Lundqvist 1966), and *Brassica* species (Hauser et al. 1998), respectively, each matching the expected pattern based on our knowledge of their divergence history and colored by the value of *m* − *M* estimated according to Table 3 or Supplemental Appendix S3. (*G*–*I*) Cartoon examples of divergence scenarios that could give rise to the isolation patterns in *A*–*C* of the same color, by matching their value of *m* − *M*. In each scenario, two parental lineages fix *D* substitutions (depicted as colored arrows), with exponentially distributed sizes (Orr 1998; see also Equation 20 of De Sanctis et al. 2023). In each case, the common ancestor is located at the ancestral optimum (labeled “Anc opt”), and the two parental populations adapt to their new optima (“P1 opt” and “P2 opt”). The gray dashed arrows in panel *I* indicate globally advantageous alleles, fixed by both parental lineages in parapatry or from shared standing variation. Note that the three orange trajectories in *G*–*I* reveal that the same pattern of isolation can be obtained in multiple ways.

This important dual role of *m* − *M* is shown in Figure 3. The upper panels, Figure 3A–C, show expected hybrid fitness for various crosses, and in various environments, which are suited to either P1 (Fig. 3A), P2 (Fig. 3C), or to neither parent (Fig. 3B). The colors in Figure 3A–C show the effects of varying *m* − *M* in each case. The middle row of panels, Figure 3D–F, then shows results from three real data sets, demonstrating how the different outcomes shown in Figure 3A–C might arise predictably under different histories of divergence. In particular, if the parental lines experienced predominantly divergent selection, pulling their phenotypes in opposing directions, then *m* (the net effect of divergence) will be large, so that *m* − *M* might be positive. This implies that average epistasis will be positive, and that all P1-specific alleles (whether ancestral or derived) will be beneficial in the P1 environment. In this case, we obtain a strong signature of ecological isolation (as in Rundle and Whitlock 2001; black lines in Fig. 3A,C) and find that admixture has the potential to increase hybrid fitness in some intermediate environments (Fig. 3B). This is the type of pattern we might observe for hybrids between ecotypes that occupy different niches. As an example, Figure 3D plots data from Rundle (2002). These data are growth rates from sticklebacks (*Gasterosteus aculeatus*) adapted to benthic and limnetic niches. The data show an abrupt decline of growth rate with limnetic (P2) ancestry. If we assume that the average benthic growth rate (the highest observed) is optimal, then we can use the estimator in the rightmost column of Table 3 to crudely calculate *m* − *M* as $5\ln w_{{\text{BC}}_{benthic\;}}^{*}-\ln w_{{\text{BC}}_{\text{limnetic}}}^{*}$, and find it to be positive as expected ($\hat{m-M}\approx 0.7$ see also Supplemental Appendix S3).

By contrast, if the parental lines have experienced ineffective selection such that they accrued random mutations, or if they have been exposed to erratically changing environments, then *m* − *M* is expected to be close to zero. In this case, there is no epistasis on average (Martin et al. 2007), such that admixture neither promotes nor reduces hybrid fitness. Instead, the main determinant of hybrid fitness is the level of heterozygosity (whose influence, from Equation 4, depends only on *M*; Simon et al. 2018), essentially because of the masking of deleterious recessives. This can lead to a pattern of heterosis, or hybrid fitness advantage, seen in the blue lines in Figure 3B. As an example, Figure 3E plots data from Lundqvist (1966), comprising kernel yields from pairwise crosses between six inbred lines of diploid rye (*Secale cereale*). In these lines, most of the divergence is likely to comprise random mutations, fixed due to inbreeding. Using the estimator based on F1 and F2 fitness from Table 3 to calculate $m-M=2\ln w_{\text{F}2}^{*}-\ln w_{\text{F}1}^{*}-\ln w_{\text{P}}^{*}$ we find a distribution of *m* − *M* overlapping with zero as expected ($\bar{m-M}=0.39$, with standard deviation 0.52 among crosses; see Supplemental Appendix S3).

Finally, whenever selection has acted to keep the parental lines close to the same optimum, then *m* will be small, and so *m* − *M* will often be negative. This means that substitutions within each parental lineage tend to compensate for each other, and hence epistasis tends to be negative. In this case, admixture is expected to decrease hybrid fitness, as the co-adapted compensatory combinations of alleles are broken up (Lynch 1991). As a result, we expect a pattern of intrinsic isolation with recombinant hybrids performing poorly in all environments (red and brown lines in Fig. 3A–C). This is the fitness pattern we would expect for hybrids between divergent species. Figure 3F shows data from Hauser et al. (1998), for hybrids between oilseed rape (*Brassica napus*) and *Brassica rapa*, a distinct species, which is both cultivated and grows as a weed alongside *B. napus*. Here, the fitness of all hybrids is lower than that of both parents, and (taking into account the variable ploidy and uniparental inheritance) we estimate *m* − *M* to be negative as expected ($\hat{m-M}=-2.64$, with log-likelihood confidence intervals (−3.4, −1.61); see Supplemental Appendix S3 for details of the estimation).

While Figure 3D–F contrast qualitatively different scenarios of divergence, for any given scenario, the pattern of isolation will depend on details of the genomic differences. For example, a given level of local adaptation might be achieved via very different genetic architectures, involving, for example, many or few changes with large or small effects, and with tight or loose linkage, and high or low levels of pleiotropy on the other traits (Wright et al. 2013; Thompson 2020). Each of these factors will affect the pattern of isolation observed.

To see this, Figure 3G shows two cases of local adaptation to two divergent optima (labeled “P1 opt” and “P2 opt”), but starting from different ancestral phenotypes (labeled “Anc opt”), and so leading to different genetic architectures (number, size, and orientation of arrows). In brown, we have large-effect substitutions that generate strong and mainly intrinsic isolation, while the smaller-effect substitutions in orange that form a shorter path between the two optima yield weaker, more environment-dependent isolation. Figure 3H shows that, even for the same phenotypic trajectories, evolution via mutations of small effect leads to less intrinsic isolation (compare brown with orange, and orange with black in panel G and H; De Sanctis et al. 2023). It follows directly that any scenario leading to larger effect substitutions is likely to enhance isolation. This observation also lies behind the “phoenix hypothesis of speciation” (Yamaguchi et al. 2022), in which isolation arises as a consequence of evolutionary rescue through fixation of large-effect alleles. We note further that tightly linked blocks of multiple changes (including inversions) may act like single, larger changes, indicating that “clumped” genetic architectures (Yeaman 2022) can also increase intrinsic isolation. Such architectures are expected to be especially common under divergence with gene flow (Yeaman 2022). Finally, Figure 3I shows how parapatry might alter results in a different way. Here, globally advantageous mutations (shifting only trait 1; dashed gray arrows) are fixed in both parental populations, such that the same orange divergence pattern could be obtained even with a more maladapted ancestor. Such a scenario was suggested to explain the patterns of local adaptation in experimentally evolved rotifer populations under different combinations of stressors (White et al. 2022). Of course, the scenarios presented in Figure 3 are highly simplified, and in reality, divergence would be unlikely to follow an exactly straight line, but the point here is to show how *m* − *M* is expected to change in predictable ways with the number, size, and orientation or pleiotropy of the alleles involved in divergent adaptation.

**Figure 4:**
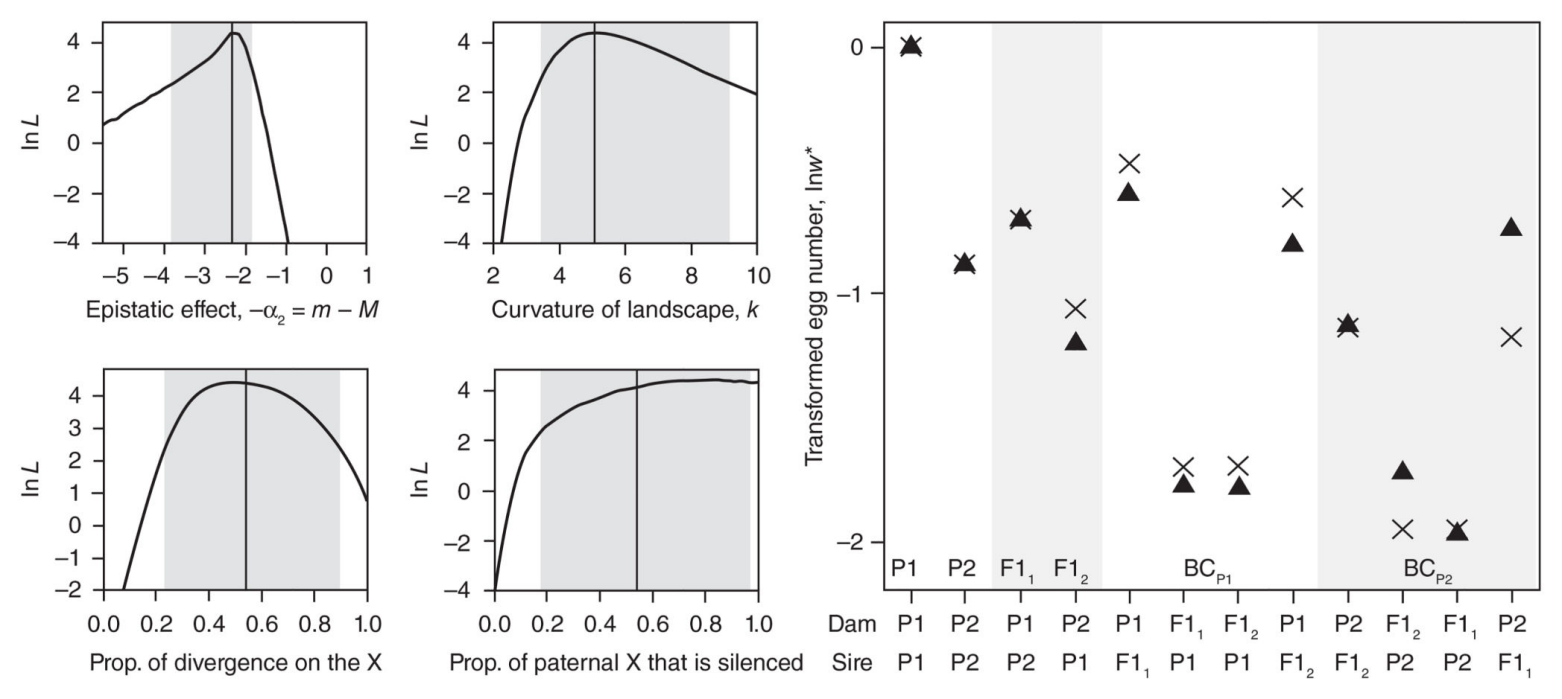
Inferring the genetic architecture of isolation from cross data. Plots show the fit of Equation 4 to data from Moran et al. (2017), reporting egg number in parental, F1 and backcross hybrids between the Australian field crickets *Teleogryllus oceanicus* (P1) and *Teleogryllus commodus* (P2). The smaller panels show the log-likelihood surfaces for the key parameters, with the maximum likelihood parameter estimates and confidence intervals shown by the vertical lines and shaded areas. The large panel compares the data (filled triangles) to the best-fit estimates (crosses) for each cross type. In each case, mean egg number is transformed to lnw^*^ using Equation 3 (main text). Full details of all analyses are given in the Supplemental Appendix S3.

Equation 4 can also be used to ask other questions about the architecture of reproductive isolation. For example, Moran et al. (2017) collected fertility data from interspecific hybrids between the Australian field crickets *Teleogryllus oceanicus* and *Teleogryllus commodus*. This species pair is a rare exception to Haldane’s rule, with hybrid fertility problems appearing solely in the XX females (Hogan and Fontana 1973; Moran et al. 2017). Data on egg number from F1 and backcross hybrids allow us to ask questions about the contribution to sterility of X-linked factors (Coyne 1992) and about the possible silencing of the paternal X (Hoy et al. 1977; Butlin and Ritchie 1989; Moran et al. 2017). This is because both factors will influence *h* and *p* in predictable ways. For example, silenced regions contribute nothing to the hybrid index, and make the homologous regions of the maternal X effectively homozygous (Simon et al. 2018). Figure 4 shows the results of fitting Equation 4, by maximum likelihood, to the data of Moran et al. (2017) (see Supplemental Appendix S3 for full details). As shown in Figure 4, the best-fit parameters included a negative value of $\hat{m-M}\approx -2$, and a high curvature $\hat{k}\approx 5$. Results also suggest that more than half of the genomic differences contributing to reduced egg number are found on the X, consistent with a large-X effect (Coyne 1992). In fact, the estimate is consistent with the size of the *Teleogryllus* X (~30% of the genome), and the X-autosome F_ST_ ratio (1.46; Moran et al. 2017, 2018; Kataoka et al. 2020). Finally, estimates suggest that the data are best fit when most of the paternal X is silenced (although with wide confidence intervals); a prediction that could be followed up by more direct methods (Rayner et al. 2021).

### What Could We Infer from a Missing Snowball?

In this section, we will compare predictions from two of the models mentioned in Table 1. Ouraim is to show how our choice of fitness landscape models can affect our inferences from hybrid data; and to show how difficult it can be to discriminate between different models. In particular, we will compare Fisher’s geometric model to the well-known and justly influential model of genetic incompatibilities introduced by Orr (1995) (see also Turelli and Orr 2000; Coyne and Orr 2004; Welch 2004; Livingstone et al. 2012; Fraïsse et al. 2014; Gavrilets 2014; Dagilis et al. 2019).

For certain kinds of data, these two models give identical predictions. For example, Simon et al. (2018) showed that Equation 4 can be derived from Orr’s model, if the parental types are optimally fit. However, the two models make very different predictions about the accumulation of incompatibilities over time. Orr’s best-known prediction is the “snowball effect,” which states that the number of genetic incompatibilities should grow at least with the square of the genomic divergence between the parents; so if two species differ at *D* sites, the number of incompatibilities should grow at least with *D*^2^. Fraïsse et al. (2016) showed via simulation that Fisher’s model does not predict the snowball effect, and instead agrees withtheverbal prediction of Muller (1942), that the number of incompatibilities will increase with *D* instead of *D*^2^. The reason for the different predictions is apparent from the third row of Table 3. The total amount of negative epistasis in any hybrid background (as quantified by the epistatic composite effect, α_2_), will depend on the total number of pairwise epistatic interactions between divergent sites

$$\left(\begin{array}{c} D\\ 2 \end{array}\right)\approx D^{2}/2$$

and on their average effect size, $\bar{\varepsilon }$. Therefore, the total amount of negative epistasis will grow with *D*^2^ as long as $\bar{\varepsilon }$ remains constant; this is, in effect, what Orr’s (1995) model assumes, and so the snowball effect follows directly. Fisher’s model, by contrast, allows $\bar{\varepsilon }$ to evolve over time, and predicts that $\bar{\varepsilon }$ will rarely remain constant. This follows because $-D^{2}\bar{\varepsilon }\approx m-M$ (Table 3); and whenever diverging populations remain close to the same optima, then *m* (the net effect of evolutionary change) will remain small, while $M\approx D\bar{\text{s}}$ will grow linearly with *D*, as more changes accrue. It follows that $\bar{\varepsilon }\approx M/D^{2}\approx \bar{\text{s}}/D$, and so $\bar{\varepsilon }$ will tend to shrink with *D* under many different divergence scenarios (Chevin et al. 2014; De Sanctis et al. 2023). In general, the assumption of a constant $\bar{\varepsilon }$ seems surprisingly difficult to generate from an explicit phenotypic model (see also Slatkin and Lande 1994; Palmer and Feldman 2009; Fierst and Hansen 2010; Kalirad and Azevedo 2017). The only exception we have found is when each new substitution has a larger effect than its predecessors, so that $\bar{\text{s}}$ gets bigger as *D* increases, which is the opposite of what is commonly observed (e.g., Imhof and Schlötterer 2001; Schoustra et al. 2009).

**Figure 5:**
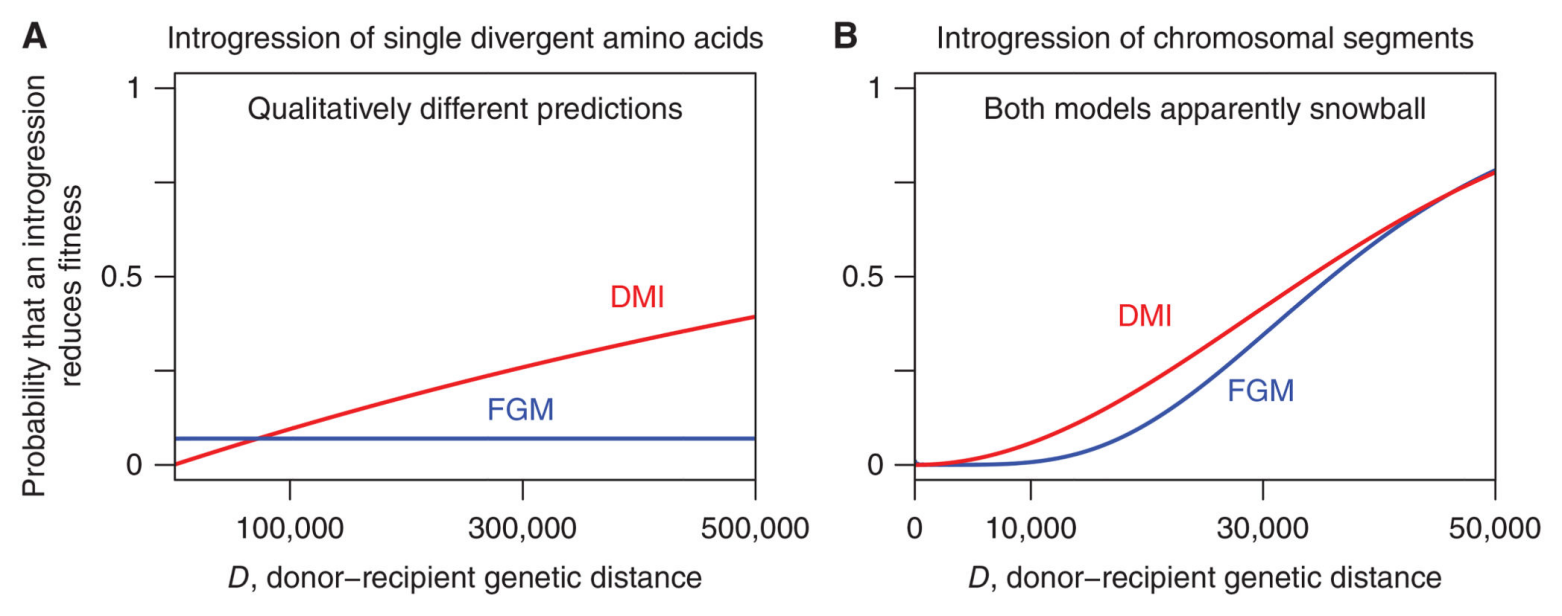
Lack of snowball for introgression of single amino acids sets model predictions apart. Analytical predictions under Fisher’s geometric model (FGM; blue lines) and the Dobzhansky–Muller incompatibility (DMI) model of Orr (1995) (red lines) are qualitatively different for the probability that single amino acid introgressions reduce fitness (*A*), whereas both models generate a very similar (apparent) snowball pattern for introgression of chromosomal segments potentially carrying multiple divergent sites (*B*). Full details and derivations are found in the Supplemental Appendix S4.

In principle, these different predictions could be tested by introgressing single divergent sites from one species to another. If a snowball effect holds, then the probability of each introgression appreciably reducing fitness should increase with the genetic distance between the do-nor and recipient species (since a linear increase in the probability implies a snowballing in the total number of incompatibilities). If, by contrast, $\bar{\varepsilon }$ declines with divergence, then the probability of an introgression reducing fitness should remain roughly constant at all levels of divergence (since this implies that the number of incompatibilities must be increasing linearly). Figure 5A compares the analytical predictions of the two models. Note, however, that the discriminatory predictions break down if the introgressed regions might contain multiple divergent sites (Fraïsse et al. 2016; see also Khatri and Goldstein 2015, 2019). This is because, under many different fitness landscape models, introgressions containing more divergent sites are more likely to be deleterious (Dagilis et al. 2022), and similarly sized introgressions will contain more divergent sites if they come from more divergent donor species. This explains the ability of Fisher’s model to generate an “apparent snowball effect” (Fraïsse et al. 2016), and this is shown in Figure 5B.

These complications do affect the interpretation of real data. For example, direct experimental tests of Orr’s model, such as the classic studies of Matute et al. (2010), Moyle and Nakazato (2010), and Wang et al. (2015), may be unable to discriminate between a real or apparent snowball effect (Fig. 5B), unless the introgressed regions are very small. Similar interpretative problems affect alternative, bioinformatic approaches (Kondrashov et al. 2002; Kulathinal et al. 2004). These studies can infer the effects of single site introgressions because identical genomic changes can arise as recurrent mutations. Such studies have not generally detected a snow-ball effect (Kondrashov et al. 2002; Kulathinal et al. 2004), which is consistent with the null predictions of Fisher’s model (Fig. 5A). However, the data are also consistent with Orr’s model, if we make additional assumptions (e.g., that all substitutions have occurred in coupled pairs (Kondrashov et al. 2002; Welch 2004; Moyle and Nakazato 2010; Presgraves 2010), or that there has been a slowdown in the substitution rate at potentially interacting sites (Gourbière and Mallet 2010; Giraud and Gourbière 2012). This example shows clearly how our choice of fitness landscape can affect our inferences about the process of between-species divergence.

### What Can We Learn from Genome Scans?

Increasingly, speciation researchers use genomic data to identify genomic regions responsible for reproductive isolation and make inferences about the forces that shape and maintain these barriers (Moran et al. 2021). Fisher’s model has been less widely used in this context, and here we will discuss some reasons for this, as well as potential directions for further research.

Most of the model’s predictions apply to gross-scale properties of the genomic data and follow directly from Equation 4. For example, the model predicts genome-wide selection for increased heterozygosity in F2-like hybrids, but not in backcross-like hybrids. This pattern was indeed observed by Simon et al. (2018) in mussels (*Mytilus edulis* and *Mytilus galloprovincialis*), and by Thompson et al. (2022) in sticklebacks (*Gasterosteus aculeatus*). As mentioned above, the same prediction can be generated by Orr’s (1995) model of Dobzhansky–Muller incompatibilities (DMI), under particular assumptions about fitness dominance (Simon et al. 2018).

Still other predictions will be shared by almost any model of negative selection against heterospecific alleles, regardless of how the background dependence is modeled (Moran et al. 2021). These robust predictions will tend to involve admixed individuals with only small fractions of introgressed ancestry that present fairly constant genomic backgrounds. This is because epistatic effects are often absorbed into additive genetic variance when minor allele frequencies are low (Hill et al. 2008). Examples include the purging of minor parent ancestryat a rate dependent on the recombination rate (Schumer et al. 2018; Moran et al. 2021; Veller et al. 2023) and clinal patterns in hybrid zones (Barton and Gale 1993; Kruuk et al. 1999).

Potential differences in model predictions might be most prominent when considering genomic outliers (e.g., in the F2; Lindtke and Buerkle 2015). For example, under a simple DMI model, selection against alleles is either negative (if the incompatible allele is also present) or neutral (if the incompatible allele is absent), so that the direction of selection against alleles will be constant across backgrounds, even if its strength can vary. Under Fisher’s model, by contrast, when the parental lineages are equally fit, both the strength and direction of selection often vary from background to background. As a result, we expect allele frequencies close to 50% in F2-like hybrids, even if there is strong overall isolation that is due to a few loci of large effect. As such, the absence of allele frequency outliers tells us relatively little about the architecture of isolation.

**Figure 6:**
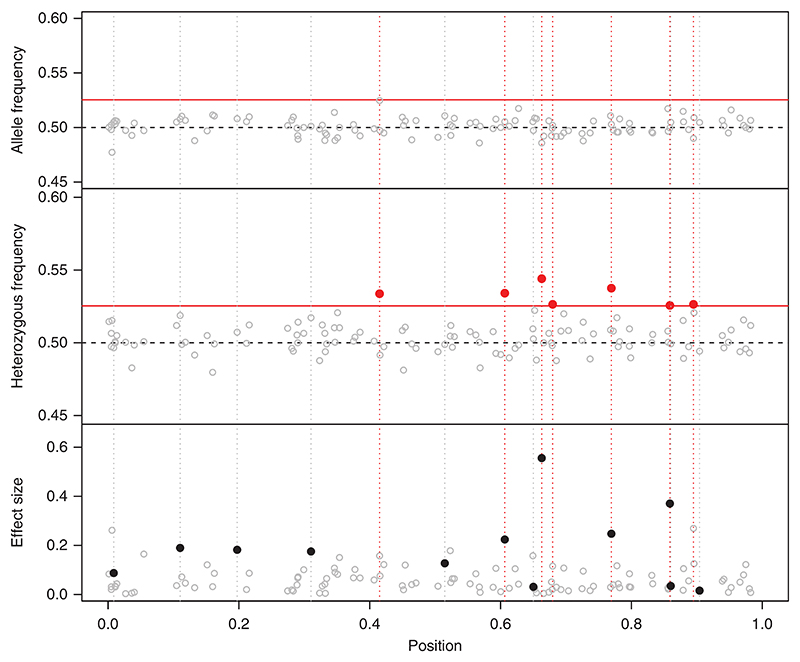
Simulated genome scans may reveal large-effect loci as outliers in heterozygosity. The *top* and *middle* panel show, for 60 freely recombining loci along the genome, the allele frequency and the proportion of heterozygous individuals among 5000 simulated F2 hybrids after viability selection. The *bottom* panel shows the effect sizes of these loci. Red points indicate outliers, exceeding the upper 99% binomial confidence interval for an expected allele or heterozygous frequency of 50%, as indicated by the horizontal red lines. Black points in the *bottom* panel show alleles fixed during the adaptation phase, defined as the period it took each population’s fitness to recover to *w* ≥ 0.90.

That said, under very specific conditions, some large-effect loci might still be detectable via their impact on heterozygosity rather than overall allele frequency. Such large-effect alleles are more likely to have fixed under adaptive divergence and are also expected to contribute disproportionally to reproductive isolation (De Sanctis et al. 2023). To illustrate this point, Figure 6 shows an example of a genome scan on 5000 simulated F2 hybrids in which seven outliers could be distinguished (red points in middle panel). Among these outliers are some of the loci with the largest effect sizes that fixed during adaptation to a novel environment under parallel directional selection (black points in bottom panel). As shown in Supplemental Appendix S5, these results can also be understood analytically by considering the expected fitness differential of hybrids carrying an allele in homozygous or heterozygous state. However, these heterozygosity peaks only appear under some parameter regimes, and are highly sensitive to many factors including the linkage map, distribution of effect sizes, and fitness landscape curvature. Therefore, it is mostly the absence of outliers of any kind that appears characteristic of highly background-dependent selection on hybrids as captured by this model, and, as things stand, we are still limited in what we can infer from genome scans. Hence, more work remainsto be done if we are to take full advantage of the signatures in genomic data to better understand the nature of genetic interactions and the interplay between divergence history and selection on hybrids.

## Closing Remarks

Fisher’s geometric model is often considered as a model of phenotypic evolution (Orr 1998; Simons et al. 2018), and it has been useful in this way in the study of speciation (e.g., Thompson 2020; Chhina et al. 2022). Here, however, we have focused instead on its role as a fitness landscape (Martin and Lenormand 2006; Martin 2014), where the phenotypic model is used simply as a way of generating predictions about the fitness effects of alleles. While, like quantitative genetics, the model’s predictions generally focus on aggregate properties, rather than the effects of individual alleles (Blanquart et al. 2014; Martin 2014), we have shown that Fisher’s model can be used to study speciation, even if we are not concerned with standard quantitative traits, and whether or not architectures are highly polygenic (Wu 2001).

We have also drawn connections between Fisher’s model and other widely used approaches (see Table 1), notably the model of DMIs (Orr 1995). Often, their predictions will be indistinguishable (see Fig. 5B; Fitzpatrick 2008; Fraïsse et al. 2016; Simon et al. 2018), but when they do differ, inferences from any single modeling approach are likely to mislead. Ultimately, of course, the utility of any model will depend on the success or failure of its predictions, that is, “the proof of the pudding is in the eating.” We can draw an extreme analogy with some machine learning algorithms, which provide useful and precise predictions without deep mechanistic insights. Closer to home, both optimization approaches and the infinitesimal model have proven useful, even though we are only starting to understand why (Grafen 2014; Martin 2014; Barton et al. 2017). In our case, despite the abstraction, we have argued that Fisher’s geometric model already provides extensive and sometimes successful predictions regarding a wide range of evolutionary phenomena and is therefore a useful tool with which to study speciation.

## Supplementary Material

Appendix

Supplementary Figure S1

Supplementary Figure S2

Supplementary Figure S3

Supplementary Figure S4

## Figures and Tables

**Table 1:** Some fitness landscape models that can be used to study speciation

Model name	Description of genotype-fitness mapping	References
Holey landscape	Each complete genotype is unfit with fixed probability	Gavrilets 1997
(Bateson-) Dobzhansky- Muller incompatibility	Each untested combination of alleles reduces fitness with fixed probability	Orr 1995
*NK* landscape	Each allele (at a total of *N* loci) has a fitness effect that depends on the allelic state at *K* other loci	Kauffman and Levin 1987
Gene regulation	Each allele has a certain binding affinity with its neighbor based on their similarity; fitness declines with the deviation of the total binding affinity from an optimal value	Johnson and Porter 2000
Fisher’s geometric	Each allele has an effect on *n* phenotypic traits; fitness declines with the distance of the trait values from their optimal values	Fisher 1930; Barton 2001

**Table 2:** Speciation questions addressed with Fisher’s geometric model

Question	General references	References using geometric model
How can reproductive isolation evolve without crossing fitness valleys?	Dobzhansky 1937; Muller 1942	Barton 1989, 2001; Mani and Clarke 1990; Chevin et al. 2014; Fraïsse et al. 2016
How is hybrid fitness affected by the history of divergence?	Mayr 1963; Dobzhansky 1946; Schluter 2000; Rundle and Nosil 2005; Gavrilets 2014	Mani and Clarke 1990; Chevin et al. 2014; Simon et al. 2018; Schneemann et al. 2020; Yamaguchi and Otto 2020; Yamaguchi et al. 2022; De Sanctis et al. 2023
What explains F1 heterosis and F2 hybrid breakdown?	Crow 1948; Edmands 1999	Barton 2001; Chevin et al. 2014; Schneemann et al. 2020, 2022
Why is the highest F1 fitness often observed at intermediate genetic distance? (optimal outbreeding and speciation clocks)	Bateson 1978; Waser 1993; Edmands 2002; Coyne and Orr 2004	Fraïsse et al. 2016; Simon et al. 2018; Schneemann et al. 2022; De Sanctis et al. 2023
How can we explain reduced fitness in heterogametic F1? (Haldane’s rule)	Haldane 1922; Muller 1942; Turelli and Orr 2000; Schilthuizen et al. 2011	Barton 2001; Fraïsse et al. 2016; Simon et al. 2018; Schneemann et al. 2022
Why do reciprocal F1 often differ in fitness? (Darwin’s corollary)	Turelli and Moyle 2007	Fraïsse et al. 2016; Schneemann et al.2022
How does uniparental inheritance affect hybrid fitness?	Mayr 1986; Vrana et al. 1998; Turelli and Moyle 2007	Fraïsse et al. 2016; Schneemann et al. 2022
Why do we sometimes observe strong complex epistasis for introgressions, or negative fitness interactions involving ≥ 3 loci?	Coyne and Orr 2004; Fraïsseet al. 2014	Barton 2001; Chevin et al. 2014; Fraïsse et al. 2016
How quickly do incompatibilities accumulate with genetic divergence? (snowball effect)	Orr 1995; Orr and Turelli 2001; Welch 2004; Gourbière and Mallet 2010; Presgraves 2010	Fraïsse et al. 2016; De Sanctis et al. 2023
How does ploidy affect outcomes of hybridization?	Wood et al. 2009; Venkataram et al. 2017	Simon et al. 2018; Kulmuni et al. 2023; H Schneemann and JJ Welch, Unpubl.
Why do double crosses often have higher fitness than F1 in tetraploids? (progressive heterosis)	Groose et al. 1989; Riddle and Birchler 2008; Washburn et al. 2019	H Schneemann and JJ Welch, Unpubl.
How do nonadditive phenotypes affect hybrid fitness?	Johnson and Porter 2000; Fierst and Hansen 2010; Khatri and Goldstein 2015; Clo et al.2021	Schneemann et al. 2020; Schneemann et al. 2022; De Sanctis et al. 2023
Under what circumstances does hybridization facilitate adaptation?	Anderson 1949; Seehausen 2004; Abbott et al. 2013; Marques et al. 2019	Kulmuni et al. 2023

**Table 3:** Interpretation and estimation of quantities in Equation 4

Quantity	Interpretaion	Simple definition	Composite effects	Estimator	Estimator when home parent optimal
*M*	Total amount of evolutionary change	$D\bar{\text{s}}$	2δ_1_	2μ(F1) − 2μ(F2) 3μ(F2) − 4μ(B) + μ(P)	$\frac{5}{4}\mu \left({\text{P}}_{H}\right)-4\mu (\text{B})$
*m*	Net effect of evolutionary change	$-\frac{1}{4}\ln w_{{\text{P}}_{A}}$	2δ_1_− α_2_	2μ(F1) − μ(P)	$-\frac{1}{4}\mu \left({\text{P}}_{\text{A}}\right)$
*m − M*	Epistatic effect	$-\left(\begin{array}{l} D\\ 2 \end{array}\right)\bar{\varepsilon }$	−α_2_	2μ(F2) − μ(F1) −μ(P) 4μ(B) − 2μ(F1)−2μ(P)	4μ(B) − 2μ(F1) – μ(P_A_) 5μ(B_H_) − μ(B_A_)
$\frac{1}{4}\left(\bar{\ln w_{{\text{P}}_{H}}^{*}}-\bar{\ln w_{{\text{P}}_{A}}^{*}}\right)$	GxE effect		α_1_ɛ	$\frac{1}{2}\left[\bar{\mu \left({\text{P}}_{\text{H}}\right)}-\bar{\mu \left({\text{P}}_{\text{A}}\right)}\right]$	$-\frac{1}{2}\bar{\mu \left({\text{P}}_{A}\right)}$

Columns 1–2: The key quantities that determine hybrid fitness under Fisher’s geometric model (Equation 4; De Sanctis et al. 2023). Third column: in the special case where *k* = 2 and one of the parents is optimal, these quantities can be expressed in terms of the average selection coefficient, $\bar{\text{s}}$, and the average pairwise epistatic effect, $\bar{\varepsilon }$ (Chevin et al. 2014). The quantities can also be translated to the composite effects of Hill (1982) (fourth column) and estimated using tools from the quantitative genetics of line crosses (fifth and sixth column; Hill 1982; Lynch 1991; Rundle and Whitlock 2001). Here μ refers to the average fitness (proxy) for hybrids of a certain type, the subscripts “H” and “A” refer to the home and away environment, and “P” and “B” refer to the average of the parental lines and reciprocal backcrosses to each parent, respectively.
