# Supplementary material for: Fisher’s Geometric Model as a Tool to Study Speciation
Source: Cold Spring Harb Perspect Biol. Author manuscript; Available in PMC 2024 Jul 2. (PMC11216183; doi:10.1101/cshperspect.a041442)
Supplement: Appendix [file EMS194048-supplement-Appendix.pdf]

Appendix of "Fisher's geometric model as a tool to study speciation"

by Hilde Schneemann, Bianca De Sanctis and John J. Welch

S1 Simulation methods for Figure 1

The simulations results presented in Figure 1 were produced following the procedure described in Schneemann et al. (2020, 2022), and De Sanctis et al. (2023) using custom scripts available at 10.5061/dryad.2bvq83bt9. In brief, we simulated allopatric divergence among populations of 100 hermaphrodite diploid individuals that were initially all identical and located at the optimum (i.e. no standing variation), which remained constant throughout the simulations. Generations were discrete and non-overlapping. Each generation individuals were picked (with replacement) as parents, with a probability proportional to their fitness. These parents formed gametes with free recombination among loci. The fitness of an individual was calculated following equations 1 and 2, but with the term in square brackets in eq. 2 multiplied by 1/2 to weaken the strength of selection following a previous simulation protocol (Schneemann et al., 2022). The gametes were subject to mutation, with a Poisson-distributed number (with mean 2) of mutations assigned to random gametes, each at a unique position (infinite sites model) such that back mutation was not possible. The additive effect (half the homozygous effect) and dominance coefficient (multiplied by the additive effect to get the heterozygous effect) for each new mutation were drawn independently for each trait from a normal and beta distribution with vanishing means, and sd=$6.85 \times 10^{-}5$, and sd=0.1667 respectively. This meant that mutations had an average selection coefficient of 0.01 in homozygous form in an optimal background, and were *on average* phenotypically additive.

We terminated the simulations once the pair of parental populations had jointly fixed 200 mutations, and used only these fixed mutations to create F1 hybrids. In making the hybrids, we randomly assigned each fixed mutations to be uniparentally expressed with a probability of 10%, such that reciprocal F1 could differ in fitness. Figure 1C shows the mean log fitness of the reciprocal F1 hybrids relative to their parents across the 100 replicate population pairs. Figure 1D shows the asymmetry in relative fitness (thin dashed lines) and squared distance (thick solid lines) between reciprocal F1 calculated as the absolute difference normalised by their mean $A(x) \equiv |x_1 - x_2|/\left(\frac{1}{2}(x_1 + x_2)\right))$, where x_1 and x_2 denote the values for the two cross directions

S2 Sunfish data reanalysis (Figure 2)

In this Appendix, we describe the simple regression method used to linearize the fitness data from centrarchid fishes, shown in Figure 2, and collated by Bolnick and Near (2005) and Bolnick et al. (2008). These data comprised reciprocal F1 crosses using ten distinct species (18 species pairs in total). The component of fitness was the hatch rate of hybrid embryos under lab conditions, divided by the hatch rate of a within-species control cross (denoted here as W_{F1} and W_{P} for the average across reciprocal F1 and parental control cross respectively, see Bolnick and Near, 2005 for full details). The evolutionary distance between each species pair in MA was taken from the dated phylogeny of Near et al. (2005). The raw data for each species pair

are shown in Figure S1A. To analyse these data using Fisher's geometric model, we first use eq. 3 to express our desired measure of log fitness ($\ln w_{\mathrm{H}}^{*}$) in terms of the fitness measurement available ($W_{\mathrm{H}}/W_{\mathrm{P}}$), noting that $\ln W_{\mathrm{H}}/W_{\mathrm{P}} = \ln W_{\mathrm{H}} - \ln W_{\mathrm{P}}$:

$$\begin{aligned} \ln w_{\mathrm{H}}^{*} &= -\left(-\ln w_{\mathrm{H}}\right)^{2/k} \\ &= -\left(-\ln W_{\mathrm{H}}/W_{\mathrm{P}} - \ln w_{\mathrm{P}}\right)^{2/k} \end{aligned} \tag{5}$$

where $\ln w_{\mathrm{P}}$ is the log *relative* fitness of the parental lines, which is relative to the (unknown) fitness for an optimal phenotype. Since there are apparently heterotic F1 in the data (Bolnick et al., 2008), the highest possible log parental fitness $\ln w_{\mathrm{P}}$ would correspond to the case where this heterotic F1 was indeed optimal, so that we have

$$\max\left(\ln w_{\mathrm{P}}\right) = \max\left(\ln W_{\mathrm{F1}}/\ln W_{\mathrm{P}}\right) \tag{6}$$

but if none of the F1 were optimal, $\ln w_{\mathrm{P}}$ could take much lower values.

Next, we make use of eq. 16 from Schneemann et al. (2022) to note that the quantity $E\left(\ln w_{\mathrm{F1}}^{*} - \frac{1}{2}\ln w_{\mathrm{P}}^{*}\right)$, where the expectation is taken across realizations of the evolutionary process, is linear in the divergence, D:

$$E\left(\ln w_{\mathrm{F1}}^{*} - \frac{1}{2}\ln w_{\mathrm{P}}^{*}\right) = \frac{\ln w_{\mathrm{P}}^{*}}{2} g^{2} - D\beta \tag{7}$$

where the slope, β, depends on the variance in the additive and dominance effects of substitutions (see Schneemann et al., 2022 for full details). The result also depends on a quantity g, which is the proportion of the divergent loci that are uniparentally inherited or expressed. While the details of sex determination and inheritance in centrarchid fishes remains unclear (Bolnick, 2009; Gomelsky et al., 2002; Roberts, 1964; López-Fernández and Bolnick, 2007), some such uniparental inheritance is implied by the observed asymmetry in the reciprocal F1 (Turelli and Moyle, 2007; Fraïsse et al., 2016).

We can now try various possible values of k and $\ln w_{\mathrm{P}}$, to find which give the highest likelihood in a linear model. As such, for each set of candidate values for k and $\ln w_{\mathrm{P}}$, we used eqs. 3 and 5 to calculate the quantity $\ln w_{\mathrm{F1}}^{*}$ for each data point. We then averaged this quantity across cross directions. Then, we regressed these transformed data onto our proxy of D, namely the evolutionary distance between the species (Near et al., 2005). We forced the regression through the origin, in effect, assuming that $\sqrt{|\ln w_{\mathrm{P}}^{*}|}g \ll 1$, so that the first term of eq. 7 could be neglected. We also used weighted regression, to account for the predicted heteroscedasticity (i.e., the fact that $\sqrt{\mathrm{Var}\left(\ln w_{\mathrm{F1}}^{*} - \frac{1}{2}\ln w_{\mathrm{P}}^{*}\right)}$ is expected to increase linearly with D. Taken together, this amounted to fitting the weighted regression:

$$E\left(y_i\right) \sim b x_i \tag{8}$$

where x_i is the evolutionary distance in MA between species pair i, the error was normally distributed

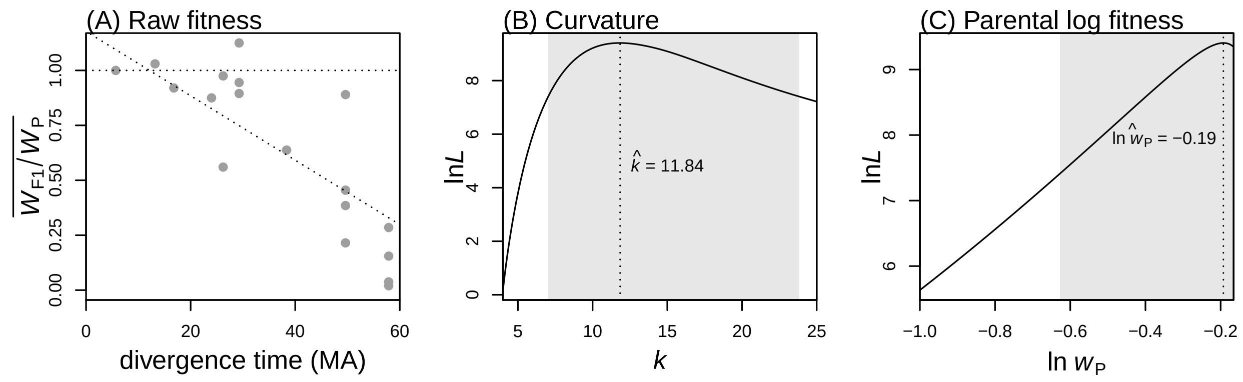

Figure S1: Parameter estimates from the sunfish data of Bolnick et al. (2008) (A) the raw data comparise the hatch rate for the reciprocal F1, divided by the rate for a control within-species cross. The F1 become progressively less fit with the divergence time of the species pair. **(B)** the log likelihood surface for the parameter k which describes the curvature of the fitness landscape. Vertical dotted line shows the Maximum Likelihood estimate of $\hat{k} = 11.84$ and the shaded area shows the confidence intervals (2 units of log likelihood). **(C)** the log likelihood surface for the fitness of the pure-species crosses, relative to the (hypothetical) optimal genotype. Confidence intervals show that we cannot reject a model where the best F1 are optimally adapted.

with variance proportional to x_i^2, and $y_i = \overline{\ln w^*_{\mathrm{F1}}} - \frac{1}{2}\ln w^*_{\mathrm{P}}$ where the bar indicates an average across the F1 cross directions.

We then found the values of k and $\ln w_{\mathrm{P}}$ (as well as the slope $\hat{b}$), that maximized the log likelihood of this linear model. As shown in Figure S1B–C, these best-fit estimates were $\hat{k} = 11.84$ and $\widehat{\ln w_{\mathrm{P}}} = -0.19$, suggesting that the parental lines had obtained $\exp[-0.19] \approx 83\%$ of the maximal possible fitness. These were the values used to produced Figure 2C–D.

Note that this regression method probably overestimated the precision of our estimates, as we made no attempt to account for phylogenetic non-independence, nor for the reuse of the same species in multiple crosses. Nevertheless, the results show that the data from centrarchid fishes are at least consistent with a table-like fitness surface (i.e., a surface with a high value of k), of the sort that can generate high fitness asymmetry, even with a high number of traits under selection.

S3 Analyses for Figures 3-4

S3.1 Data reanalyses from Figure 3D-F

In this section of the Appendix, we briefly describe the analysis of the data plotted in Figure 3D-F. For the stickleback data from Rundle (2002) (Fig. 3D), we used growth rate measurements in the littoral environment as our proxy of relative fitness. We normalised these with the mean of the benthic individuals, such that the benthics are *on average* optimal (i.e. $\ln w_{\mathrm{benthic}} = 0$), and we can estimate m directly from the fitness of the limnetics as $\ln w_{\mathrm{limnetic}} = -4m$. The mean and 2.5-97.5% quantiles of log relative fitness for each category of individual (benthic, backcross to benthic, backcross to limnetic, and limnetic) is plotted with the dots and vertical lines in Figure 3D. In doing our normalisation, we also assume that $k = 2$, which might be a fair approximation for within-species hybrids. We can now calculate $m - M$ as

$\widehat{m-M} = 5 \ln w_{\mathrm{BC}_{\mathrm{benthic}}} - \ln w_{\mathrm{BC}_{\mathrm{limnetic}}}$ (see Table 3). If we had more data, it would be preferable to fit k and the optimal growth rate value, as demonstrated in the other case studies.

For the rye analysis, we used total kernel yield as our proxy of absolute fitness. We analysed the data for the diploid inbred lines and their hybrids from the experiments conducted in 1959 by Lundqvist (1966). We normalised these measurements by the maximum yield observed in this study to obtain an estimate of relative fitness w. We computed $m - M$ for each cross (again assuming $k = 2$) as $\widehat{m-M} = 2 \ln w_{\mathrm{F2}} - \ln w_{\mathrm{F1}} - \ln w_{\mathrm{P}}$, noting that this analysis does not take into account potential maternal effects.

For the analysis of the *Brassica* hybrids, we used the combined fitness measurement reported in Table 1 of Hauser et al. (1998). As this dataset contains reciprocal backcrosses to both parental species, we need to model uniparental inheritance or expression (using g again to denote the proportion of the genome that is uniparentally inherited or expressed, in this case e.g. linked to the mitochondria or chloroplast) to account for the fitness asymmetry between the reciprocals. In particular, we define the hybrid index and heterozygosity as a weighted sum over the biparentally and maternally expressed portions of the genome (see Table S1). As these are interspecific hybrids between more divergent parents, we would also like to estimate k rather than use the $k = 2$ approximation. To do this we will again use eqs. 3 and 5 to transform the data for different possible values of k. Assuming that the value reported for *B. napus* (denoted here as P1) represents optimal fitness, we have $\ln w^*_{\mathrm{P1}} = 0$ and $m = \frac{1}{4} \ln w^*_{\mathrm{P2}}$. Setting m in this way, we can then treat $m - M$ as a single parameter, such that eq. 4 becomes:

$$\widehat{\ln w^*_{\mathrm{H}}} \;=\; h \;\ln w^*_{\mathrm{P2}} \;+\; 4h\,(1-h)\,(m-M) \;\;-\; p\left((m-M) + \tfrac{1}{4}\ln w^*_{\mathrm{P2}}\right) \tag{9}$$

which depends only on k, $m - M$ and g (following Table S1). We use a maximum likelihood approach to find the values of these parameters that provide the best fit of our model in eq. 9 to the data. To do this we assume that, for any given cross, $\ln w^*_{\mathrm{H}} - \widehat{\ln w^*_{\mathrm{H}}}$ is normally distributed with zero mean and a fixed variance which is also estimated to maximize the likelihood. For every combination of 40 possible values for $k, m - M$, and g in the range $[2, 20]$,$[-10, 0]$, and $[0, 1]$ respectively, we fit a linear model with no parameters (i.e. $\ln w^*_{\mathrm{H}} - \widehat{\ln w^*_{\mathrm{H}}} \sim 0$) to obtain a log-likelihood value for the set of candidate values.

The marginal log-likelihood surfaces for each of these parameters are plotted in Figure S2. While there are some potential issues with this estimation procedure, as will be discussed below, we find that this dataset supports a value of $k > 2$ and $m - M < 0$ as we might expect for distinct species growing alongside each other in the same habitat. The data also suggest that only a minor fraction of the genome is uniparentally inherited or expressed ($g < 0.5$), consistent with the relatively small size of the mitochondrial and chloroplast genome relative to the biparentally expressed nuclear genome. While *B. napus* is an allotetraploid carrying a second subgenome more diverged from that of *B. rapa*, the two subgenomes have been shown to segregate mostly independently during meiosis in hybrids (Leflon et al., 2006) and hence we can consider the absence of this second subgenome as the *B. rapa* allele such that predictions for the hybrid index and heterozygosity remain unchanged.

Table S1: Hybrid index h and heterozygosity p for the *Brassica* cross types The hybrid index and heterozygosity are both functions of the proportion of divergence that is uniparentally inherited or expressed g including loci on the mitochondria and chloroplast genome.

Cross type	Dam	Sire	h	p
P	*B. rapa*	*B. rapa*	0	0
BC_{P1}	*B. rapa*	F1	$(1-g)/4$	1/2
BC_{P1}	F1	*B. rapa*	$(1+g)/4$	1/2
F2	F1	F1	1/2	1/2
BC_{P2}	F1	*B. napus*	$(3-g)/4$	1/2
BC_{P2}	*B. napus*	F1	$(3+g)/4$	1/2
P	*B. napus*	*B. napus*	1	0

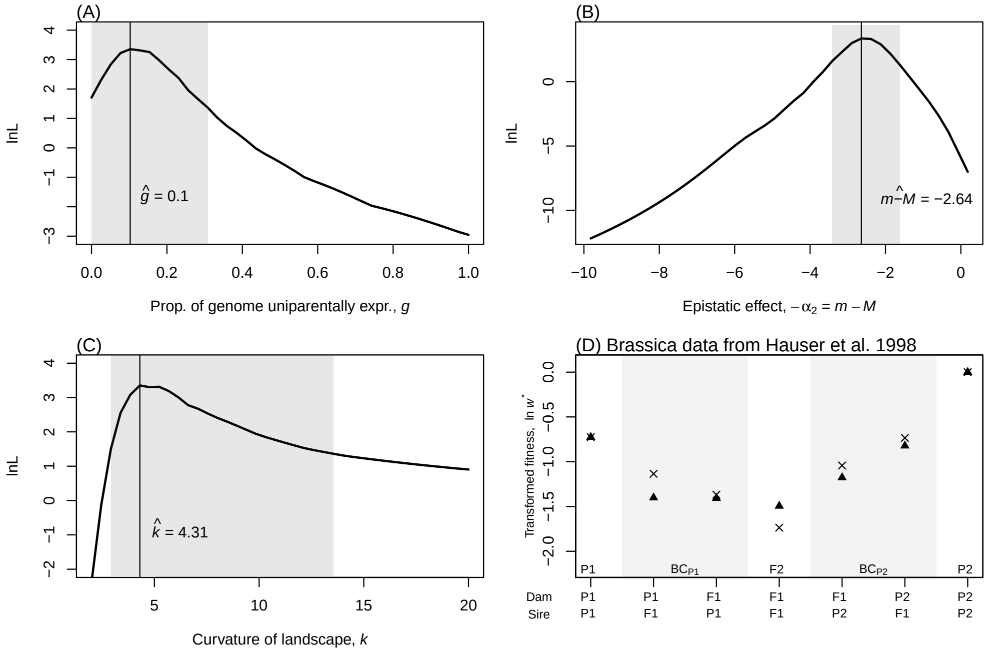

Figure S2: Parameter estimates from the *Brassica* data by Hauser et al. (1998) Plots show the fit of eq. 9 to data from Hauser et al. (1998), reporting a combined fitness measure of parental, F2 and backcross hybrids between oilseed rape *Brassica napus* (P1) and the weed *Brassica rapa* (P2). **(A)-(C)** The log likelihood surfaces for the parameters g, $m-M$, and k which describe the proportion of the genome that is uniparentally inherited or expressed, the epistatic effect, and the curvature of the fitness landscape respectively. Vertical lines show the Maximum Likelihood estimates and the shaded areas show the confidence intervals (2 units of log likelihood). **(D)** Comparison of the $\ln w^*$ values of the data (filled triangles) to the best-fit estimates (crosses) for each cross type.

Table S2: Expected hybrid index h and heterozygosity p for the *Teleogryllus* cross types. The hybrid index and heterozygosity are both functions of the proportion of divergence that is linked to the X chromosome, denoted $0 \leq g \leq 1$, and the proportion of the paternal X chromosome that is silenced, denoted $0 \leq \pi \leq 1$.

Cross type	Dam	Sire	h	p
P1	P1 (*T. oceanicus*)	P1	0	0
P2	P2 (*T. commodus*)	P2	1	0
$F1_1$	P1	P2	$(1-g\pi)/2$	$1-g\pi$
$F1_2$	P2	P1	$(1+g\pi)/2$	$1-g\pi$
BC_{P1}	P1	$F1_1$	$(1-g)/4$	$(1-g)/2$
BC_{P1}	$F1_1$	P1	$(1+g\pi)/4$	$(1-g\pi)/2$
BC_{P1}	$F1_2$	P1	$(1+g\pi)/4$	$(1-g\pi)/2$
BC_{P1}	P1	$F1_2$	$(1+g)/4-g\pi/2$	$(1+g)/2-g\pi$
BC_{P2}	P2	$F1_2$	$(3+g)/4$	$(1-g)/2$
BC_{P2}	$F1_2$	P2	$(3-g\pi)/4$	$(1-g\pi)/2$
BC_{P2}	$F1_1$	P2	$(3-g\pi)/4$	$(1-g\pi)/2$
BC_{P2}	P2	$F1_1$	$(3-g)/4+g\pi/2$	$(1+g)/2-g\pi$

S3.2 Reanalysis of *Teleogryllus* data (Figure 4)

For the reanalysis of the data of Moran et al. (2017), shown in Figure 4, we extended the procedure described above to analyse the *Brassica* data. In this case, the fitness proxy was the expected number of eggs produced (the proportion of matings that produced eggs, multiplied by the mean number of eggs produced by the successful matings). We fit eq. 9 to these data as described, but now altered the expected values of h and p to include a new parameter, $0 \leq \pi \leq 1$ which represents the proportion of the paternal X that is silenced (and treating effectively hemizygous X-linked loci as if they were homozygous). The relevant values for the crosses are shown in Table S2 (We note here that Moran et al., 2017 also reported data for the second backcross, which we did not use, because of anomalous results for the pure-species controls in this generation).

The analysis reported in Figure 4 makes two assumptions that are especially questionable: (1) that mean egg number for pure-species *Teleogryllus oceanicus* matings represents the optimal value; and (2) that we can safely approximate the mean of a function with the function of a mean (such that $E(\ln w^*) \approx -(-\ln \bar{w})^{2/k}$). (Note that if one had the raw data for each individual, this second assumption would not be necessary as the averaging could simply be done after the transformation of the data). We therefore carried out two analyses to see if our qualitative conclusions were robust to relaxing these assumptions. Both assumed a $k=2$ landscape, but took a more principled approach to averaging.

For the first such method, we reanalyzed the data equating egg number with log absolute fitness instead of absolute fitness, such that

$$\ln w^* \equiv \overline{W} - W_0. \tag{10}$$

where $\overline{W}$ is the mean egg number for a given cross, and W_0 is the optimal egg number (now treated as a free parameter). We then equated the net effect of evolutionary change with $m = \frac{1}{4}(\ln w^*_{P1} + \ln w^*_{P2})$,

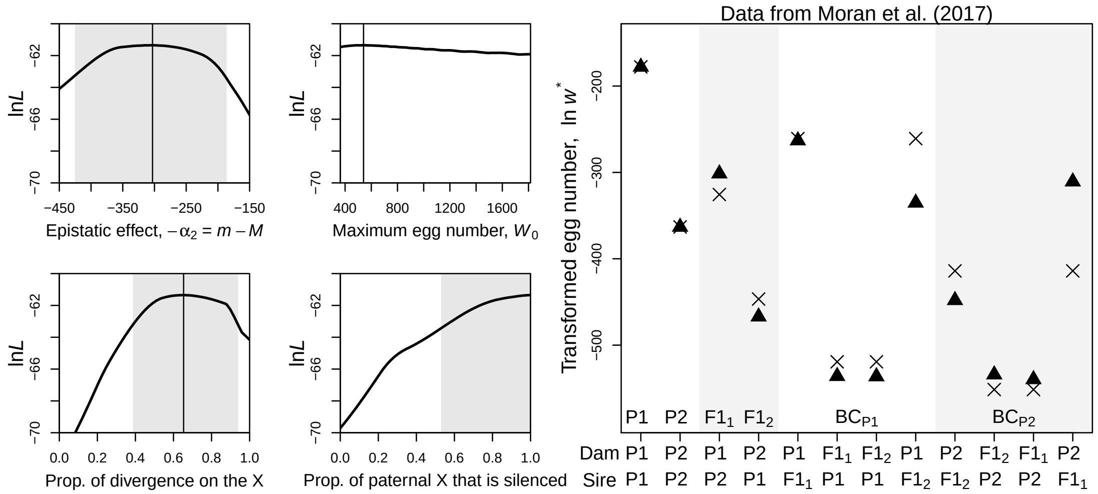

Figure S3: Reanalysis of the *Teleogryllus* data with equation 10. All other details match Figure 4

which allows for the fact that both parents may be maladapted, but assumes that they are maladapted in orthogonal phenotypic dimensions (Simon et al., 2018; Schneemann et al., 2022).

Figure S3 shows the result of this modified procedure. Most importantly, we find that the results regarding X silencing are relatively robust, as the best-fit model includes a completely inactivated paternal X, and a model with completely biparental expression is strongly rejected. Furthermore, we obtain a quantitatively similar estimate for the proportion of X-linked divergence, and also a negative albeit much more extreme estimate for the epistatic effect, α_2. One difference with the previous procedure is that we do not assume either parental species is optimal. As a result, we obtain an estimate for the maximum egg number (W_0). This estimate is reasonably close to its lower bound, which we set at $\min(W_0) = \overline{W}_{\mathrm{P1}}$, since the mean egg number for *T. oceanicus* was the highest observed in the dataset. However, the likelihood surface is very flat indicating that this parameter cannot be estimated with confidence.

For our second approach, we exploited the fact that Moran et al. (2017), as well as reporting the mean number of eggs for each cross, $\overline{W}$, also reported the variance in egg number, V_W (calculable from the proportion of matings yielding eggs, and the mean and standard deviation in egg number for the successful matings). We can therefore estimate the squared distance form the optimum if we make assumptions about the distribution of egg number for a cross. Let us begin by defining the mean and variance in *relative* fitness as

$$\bar{w} \equiv \overline{W}/W_0$$
$$V_w \equiv V_W/W_0^2 \tag{11}$$

where W_0, as above, is the (unknown) optimal fitness. We then assume that $0 \leq w \leq 1$ is beta-distributed, so that $E(\ln w) = \psi(\alpha) - \psi(\alpha+\beta)$ where $\psi(\cdot)$ is the digamma function, and α and β are the shape parameters of the beta distribution. These shape parameters can, in turn, be estimated from the observed means and variances. As such, we used the following transformation:

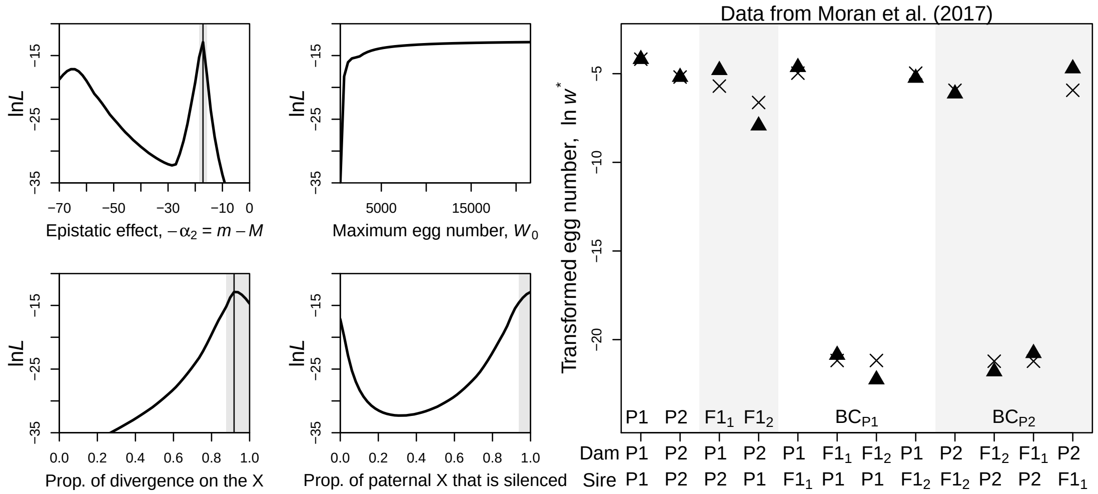

Figure S4: Reanalysis of the *Teleogryllus* data with equations 11–13. All other details match Figure 4

$$\ln w^* \equiv \psi(\hat{\alpha}) - \psi(\hat{\alpha} + \hat{\beta}). \tag{12}$$

where

$$\begin{aligned} \hat{\alpha} &\equiv \bar{w}(\bar{w}(1-\bar{w})/v_w - 1) \\ \hat{\beta} &\equiv (1-\bar{w})(\bar{w}(1-\bar{w})/v_w - 1) \end{aligned} \tag{13}$$

Figure S4 shows the results of refitting the data from Moran et al. (2017) using equations 11–13. Compared to the previous analysis, the fit of the predictions to the data is better for some of the backcrosses, but is slightly worse for the F1s. Moreover, the best-fit maximum number of eggs becomes unrealistically large under this procedure, but can still not be estimated with any precision. The proportion of X-linked divergence is also greater than in the other two analyses of these data. For the epistatic effect and the proportion of the paternal X that is silenced, we now find bimodal likelihood surfaces, where the second, lower peak for $m - M$ corresponds to the lower peak of no paternal X silencing. This suggests that there might be two alternative interpretations of the patterns in this dataset. Nevertheless, this analysis continues to support a model with (nearly) complete paternal X silencing as providing the best fit to the data.

S4 Derivations for Figure 5

In this section of the Appendix, we derive the curves plotted in Figure 5. We note that panels A and B plot two different quantities. Figure 5A plots the probability that the introgression of an allele causing a single amino acid change from one species to another will cause an appreciable loss of fitness. This probability is denoted P_{single}. We note that P_{single} is relevant to interpreting bioinformatic studies, as pioneered by

Kondrashov et al. (2002), in which mutations with known deleterious effect in one species are identified as the wild-type in a second species. In such studies, the "introgressions" always involve a single divergent site. By contrast, Figure 5B corresponds to introgressions from one species to another, of a fixed proportion of the genome (denoting the proportion as $0 < h \ll 1$, because it constitutes the hybrid index of the introgression line). Such introgressions could involve multiple divergent sites, and so the probability that they cause an appreciable loss in fitness is denoted P_{multi}. This quantity is relevant to interpreting studies such as Matute et al. (2010), which utilized crosses involving balancer chromosomes and deficiency lines. The relevance of P_{single} and P_{multi} to different types of empirical study explains the different range of D values used on the x-axes of Figure 5, but these choices do not otherwise affect the conclusions drawn.

We now note that both P_{single} and P_{multi} are related to a quantity p_d, which denotes the probability that a genomic segment containing exactly d divergent sites will lead to a substantial reduction in fitness when introgressed from one species to another. In particular, we have

$$P_{single} = p_1$$

and for randomly distributed genomic changes, we have

$$P_{multi} = \sum_{d=0}^{D} h^D (1-h)^{D-d} \binom{D}{d} p_d$$

We now ask how these two quantities vary with D under the two fitness landscape models.

S4.1 Results under the standard DMI model

Under the assumption of Orr's (1995) model, each introgressed site might interact negatively with each of the remaining $D-d$ sites. However, if the loci are biallelic, then with a probability 1/2, the pair of alleles will have appeared together at high frequency during the divergence process, implying that they are not incompatible. If they have not appeared together, and are therefore an "unproven" combinations of alleles, then, by assumption, they create an incompatibility with some probability q. It follows therefore, that the probability of obtaining an incompatibility by introgressing d divergent sites, is simply:

$$p_d = 1 - (1-q/2)^{d(D-d)} \tag{14}$$

It then follows immediately, that

$$\begin{aligned} P_{single} &= 1 - (1-q/2)^{D-1} \\ &\approx 1 - e^{-Dq/2} \end{aligned} \tag{15}$$

Equation 15 predicts that P_{single} will increase linearly with D when qD is small, and it is plotted as the

red line in Figure 5A, assuming $q = 2 \times 10^{-6}$ (Orr, 1995). For multi-site introgressions (Figure 5B), we can use $p_d \approx 1 - e^{-qdD/2}$, then approximate the binomial distribution with a Poisson, and $e^{-Dq/2} \approx 1 - Dq/2$. This yields

$$P_{multi} \approx 1 - e^{-D^2 hq/2} \tag{16}$$

Equation 16 is plotted as the red line in Figure 5B, assuming that introgressions comprised a fraction $h = 3 \times 10^{-4}$ of the genome. Note also the dependency on D^2, which defines the true snowball effect.

S4.2 Results under Fisher's geometric model

To derive equivalent results under the phenotypic model, we begin by assuming that both the donor and recipient species are well adapted to current environmental conditions, so that their phenotypes are close to the optimum. In this case, we can model the D phenotypic effects on each of their n traits as an independent Brownian bridge (a tethered Brownian motion) starting and ending at the optimum (Schneemann et al., 2020). This implies that the effects on any single trait of introgressing d randomly-chosen alleles from one species to another will be normally distributed with mean 0 and variance $d\left(1 - d/D\right)/n$ (where we have, without loss of generality, scaled the trait measurements by the typical size of a single introgression). It follows that the transformed fitness of the introgression line will be distributed as

$$\ln w^* \sim -\frac{d(1 - d/D)}{n} x_n$$

where x_n is standard Chi-squared distributed variable, with n degrees of freedom. Let us now define as $\ln w^*_{\mathrm{I}}$ the threshold log fitness that would lead us to score the introgression as an incompatibility. It follows that the probability of observing an incompatibility by introgressing d divergent sites, is

$$\begin{aligned} p_d = \Pr\left(\ln w^* < \ln w^*_{\mathrm{I}}\right) &= \Pr\left(x_n > \frac{-\ln w^*_{\mathrm{I}} n}{d(1 - d/D)}\right) \\ &= 1 - P\left(\frac{n}{2}, \frac{n}{2}\frac{-\ln w^*_{\mathrm{I}}}{d(1 - d/D)}\right) \end{aligned}$$

where $P(.,.)$ is the regularized gamma function: $P(a, z) \equiv \gamma(a, z)/\Gamma(a)$, which is the cumulative density of the Chi-squared distribution. It follows therefore that

$$P_{single} = 1 - P\left(\frac{n}{2}, \frac{n}{2}\frac{-\ln w^*_{\mathrm{I}}}{1 - 1/D}\right) \tag{17}$$

For large D, eq. 17 is independent of D, so that the probability is a constant. This is plotted as the blue lines in Figure 5A, after assuming that $n = 20$ and $\ln w^*_{\mathrm{I}} = -3/2$. For multi-site introgressions, the blue line in Figure 5B plots

$$P_{multi} = 1 - \sum_{d=0}^{D} h^D (1-h)^{D-d} \binom{D}{d} P\left(\frac{n}{2}, \frac{n}{2} \frac{-\ln w_{\mathrm{I}}^*}{d(1-d/D)}\right) \tag{18}$$

which was calculated numerically, after choosing $\ln w_{\mathrm{I}}^* = -10$ for ease of visualisation. In general, the relevant value of $\ln w_{\mathrm{I}}^* = -10$ would depend on the shape of the fitness function, and on the experimental protocol (e.g. the subjective choice of what is considered an incompatibility). For a high-k landscape (§2.1), $\ln w_{\mathrm{I}}^* = -1$ would be a natural choice, however, the data of Matute et al. (2010) showed finer-grained differences in fitness. We note finally, that Khatri and Goldstein (2015, 2019) presented a model of incompatibilities that is qualitatively similar to Fisher's model, but with non-additive effects on phenotype, inspired by the biology of transcription-factor and polymerase binding. Khatri and Goldstein (2015, 2019) showed that the model can generate a snowball effect under some conditions, although the incompatibilities involve multiple divergent sites in the relevant sequences, so in the terminology used here, this could be an "apparent snowball".

S5 Analyses for Figure 6

S5.1 Analytical prediction

To predict the signature of large-effect loci in genome scans of F2 hybrids between equally fit parental lineages, we need to find the expected difference in fitness between hybrids that are heterozygous or homozygous at a given focal site i with phenotypic effect a_{ij} on trait j. We will denote the log fitness of the hybrids heterozygous for this site as $\ln w_{\mathrm{H},12}^*$ and the log fitness of the hybrids homozygous for the P1 and P2 allele as $\ln w_{\mathrm{H},11}^*, \ln w_{\mathrm{H},22}^*$ respectively. Using eq. 56 of De Sanctis et al. (2023), we find

$$E\left(\ln w_{\mathrm{H},12}^* - \frac{\ln w_{\mathrm{H},11}^* + \ln w_{\mathrm{H},22}^*}{2}\right) = 2\frac{M + \sum_{j=1}^{n} a_{ij}^2}{D} + 4\frac{\sum_{j=1}^{n} a_{ij} \sum_{k=1;k\neq i}^{D} a_{kj}}{D\,(D-1)} + 2\frac{m-M}{D\,(D-1)} \tag{19}$$

$$\approx 2\frac{M+s}{D} \qquad D >> 1 \tag{20}$$

where m and M are defined across the non-focal sites. Eq. 19 shows that the exact result depends on the orientation of the substitution with respect to the phenotypic divergence between the parents. Nevertheless, if there are many divergent loci, we obtain the simple result of eq. 20 which clearly shows how the fitness advantage of the heterozygote and consequently the level of excess heterozygosity at this locus is expected to increase with the effect size of the allele, i.e. its selection coefficient s. This shows that large-effect loci provide the highest fitness when carried in heterozygous state, leaving a signature of excess heterozygosity across hybrid individuals and explaining the pattern observed in Figure 6.

S5.2 Heterozygosity scan on simulated F2s

Figure 6 shows a heterozygosity scan on 5000 simulated F2 hybrid genomes. These were simulated using the same procedure as described above in section S1, except the population size was 1000, the number of traits was $n = 20$, and the landscape curvature was $k = 6$. There was an expected number of 0.2 new mutations per generation. These new mutations were phenotypically additive, with a random orientation in n-dimensional trait space, and a total magnitude drawn from an exponential distribution. The mean of this distribution was set such that the mean selection coefficient in an optimal background was $\bar{s} = 0.1$. The optimum was displaced on trait 1 such that the common ancestor was maladapted, with a distance of 1 phenotypic unit, such that the ancestral relative fitness was $w \approx 0.6$. These parameter settings were chosen in order to maximize variation in the size of substitutions. Simulations were terminated as soon as one parental population had fixed 60 substitutions, resulting in 117 divergent sites in total.

To generate the F2 generation, gametes were formed with free recombination among loci. F2 individuals were then subjected to viability selection, such that each survived with a probability proportional to its fitness, resulting in 2610 survivors. The allele frequency among the survivors, and the proportion of survivors that is heterozygous for a given locus is plotted along the genome in the top and middle panel of Figure 6 respectively. The bottom panel shows the effect size of each locus as the sum of the squared homozygous effect across the 20 traits. The position of the loci across the x-axis is arbitrary. The loci for which the heterozygous frequency exceeded 0.525%, the upper 99% binomial confidence interval using the exact Pearson-Klopper method (Clopper and Pearson, 1934), were identified as outliers and are colored in red. The vertical red dotted lines connect these outliers to their corresponding effect size and allele frequency. The 12 substitutions that fixed as the parental populations first approached the optimum to within .2 squared distance are marked in black in the bottom panel, and are connected by vertical dotted lines to their corresponding allele and heterozygous frequencies.

References

Bolnick, D. I. (2009). Hybridization and Speciation in Centrarchids. In Cooke, S. J. and Philipp, D. P., editors, *Centrarchid Fishes*, pages 39–69. Wiley-Blackwell, Oxford, UK.

Bolnick, D. I. and Near, T. J. (2005). Tempo of hybrid inviability in centrarchid fishes (Teleostei: Centrarchidae). *Evolution*, 59(8):1754–1767.

Bolnick, D. I., Turelli, M., López-Fernández, H., Wainwright, P. C., and Near, T. J. (2008). Accelerated mitochondrial evolution and "Darwin's corollary": Asymmetric viability of reciprocal F1 hybrids in centrarchid fishes. *Genetics*, 178(2):1037–1048.

Clopper, C. J. and Pearson, E. S. (1934). The use of confidence or fiducial limits illustrated in the case of the binomial. *Biometrika*, 26(4):404–413.

De Sanctis, B., Schneemann, H., and Welch, J. J. (2023). How does the mode of evolutionary divergence affect reproductive isolation? *Peer Community J.*, 3(e6).

Fraïsse, C., Gunnarsson, P. A., Roze, D., Bierne, N., and Welch, J. J. (2016). The genetics of speciation: Insights from Fisher's geometric model. *Evolution*, 70(7):1450–1464.

Gomelsky, B., Mims, S. D., Onders, R. J., and Bean, W. B. (2002). Hormonal Sex Reversal and Evidence of Female Homogamety in Black Crappie. *North American Journal of Aquaculture*, 64(1):66–69.

Hauser, T. P., Jørgensen, R. B., et al. (1998). Fitness of backcross and F2 hybrids between weedy *Brassica rapa* and oilseed rape (*B. napus*). *Heredity*, 81(4):436–443.

Khatri, B. S. and Goldstein, R. A. (2015). Simple biophysical model predicts faster accumulation of hybrid incompatibilities in small populations under stabilizing selection. *Genetics*, 201(4):1525–1537.

Khatri, B. S. and Goldstein, R. A. (2019). Biophysics and population size constrains speciation in an evolutionary model of developmental system drift. *PLoS Computational Biology*, 15(7):e1007177.

Kondrashov, A. S., Sunyaev, S., and Kondrashov, F. A. (2002). Dobzhansky–Muller incompatibilities in protein evolution. *Proceedings of the National Academy of Sciences*, 99(23):14878–14883.

Leflon, M., Eber, F., Letanneur, J. C., Chelysheva, L., Coriton, O., Huteau, V., Ryder, C. D., Barker, G., Jenczewski, E., and Chèvre, A. M. (2006). Pairing and recombination at meiosis of *Brassica rapa* (AA) x *Brassica napus* (AACC) hybrids. *Theor Appl Genet*, 113(8):1467–1480.

Lundqvist, A. (1966). Heterosis and inbreeding depression in autotetraploid rye. *Hereditas*, 56(2-3):317–366.

López-Fernández, H. and Bolnick, D. I. (2007). What causes partial f1 hybrid viability? incomplete penetrance versus genetic variation. *PLoS ONE*, 2(12):1–8.

Matute, D. R., Butler, I. A., Turissini, D. A., and Coyne, J. A. (2010). A test of the snowball theory for the rate of evolution of hybrid incompatibilities. *Science*, 329(5998):1518–1521.

Moran, P. A., Ritchie, M. G., and Bailey, N. W. (2017). A rare exception to Haldane's rule: Are X chromosomes key to hybrid incompatibilities? *Heredity*, 118(6):554–562.

Near, T. J., Bolnick, D. I., and Wainwright, P. C. (2005). Fossil calibrations and molecular divergence time estimates in centrarchid fishes (Teleostei: Centrarchidae). *Evolution*, 59(8):1768–1782.

Orr, H. A. (1995). The population genetics of speciation: The evolution of hybrid incompatibilities. *Genetics*, 139:180–185.

Roberts, F. L. (1964). A chromosome study of twenty species of centrachidae. *Journal of Morphology*, 115(3):401–417.

Rundle, H. D. (2002). A test of ecologically dependent postmating isolation between sympatric sticklebacks. *Evolution*, 56(2):322–329.

Schneemann, H., Munzur, A. D., Thompson, K. A., and Welch, J. J. (2022). The diverse effects of phenotypic dominance on hybrid fitness. *Evolution*, 76(12):2846–2863.

Schneemann, H., Sanctis, B. D., Roze, D., Bierne, N., and Welch, J. J. (2020). The geometry and genetics of hybridization. *Evolution*, 74(12):2575–2590.

Simon, A., Bierne, N., and Welch, J. J. (2018). Coadapted genomes and selection on hybrids: Fisher's geometric model explains a variety of empirical patterns. *Evolution Letters*, 2(5):472–498.

Turelli, M. and Moyle, L. C. (2007). Asymmetric postmating isolation: Darwin's corollary to Haldane's rule. *Genetics*, 176:1059–1088.
