# Supplementary figures and images for: Fisher’s Geometric Model as a Tool to Study Speciation
Source: Cold Spring Harb Perspect Biol. Author manuscript; Available in PMC 2024 Jul 2. (PMC11216183; doi:10.1101/cshperspect.a041442)

(A) Raw fitness

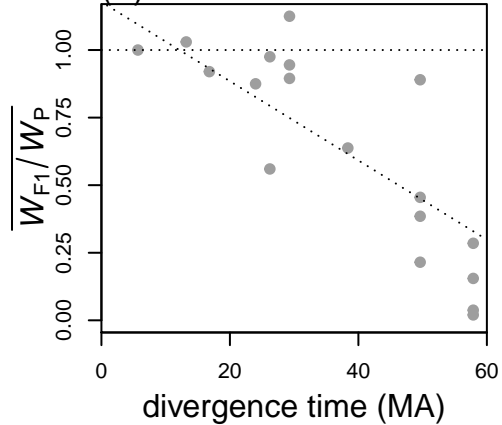

(B) Curvature

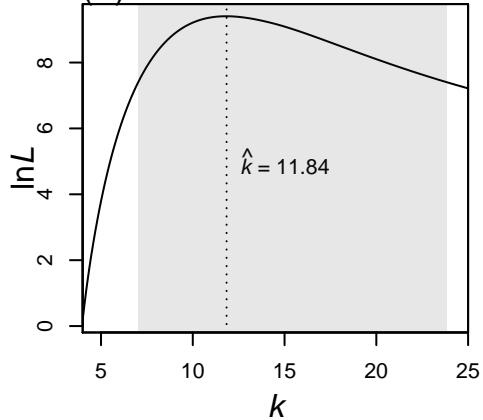

(C) Parental log fitness

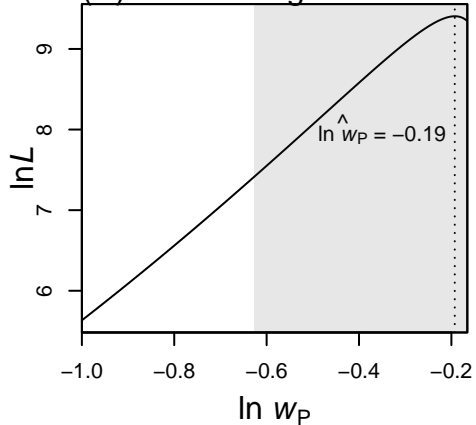

Supplement: Supplementary Figure S1 [file EMS194048-supplement-Supplementary_Figure_S1.pdf]

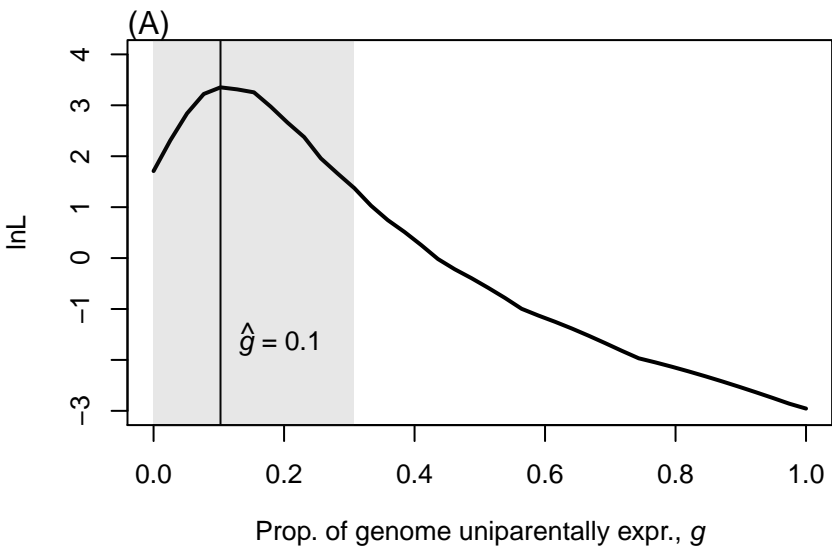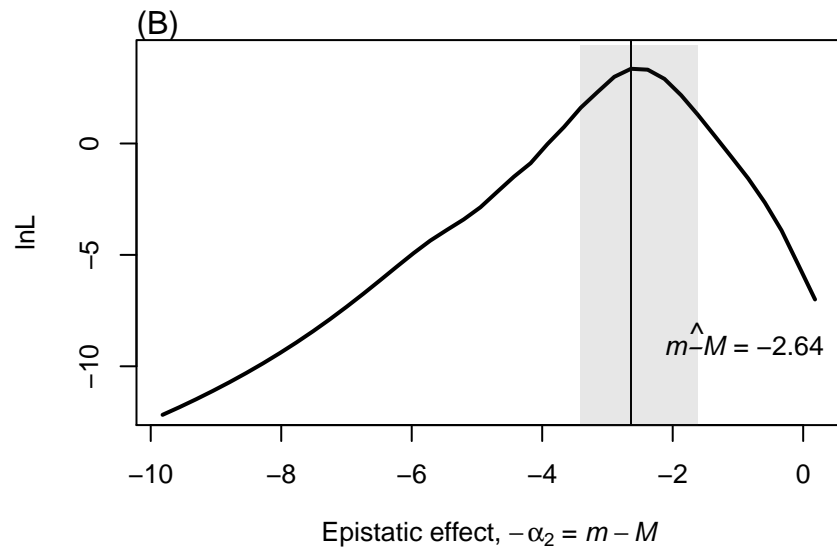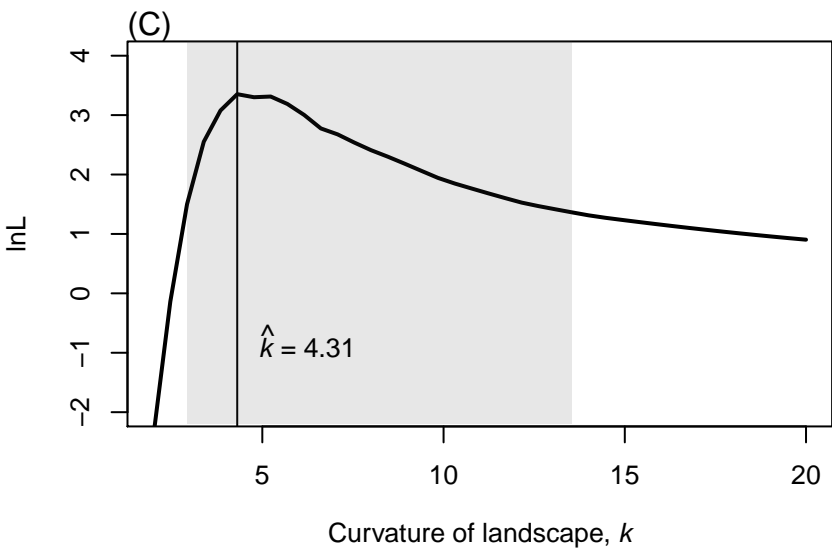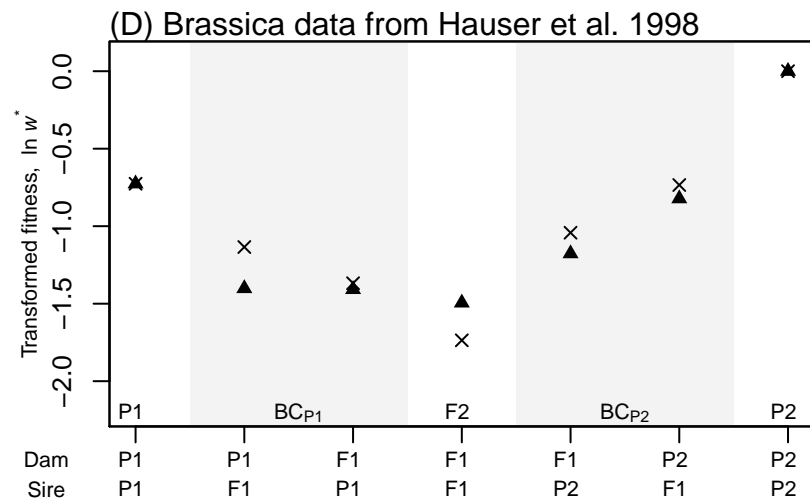

Supplement: Supplementary Figure S2 [file EMS194048-supplement-Supplementary_Figure_S2.pdf]

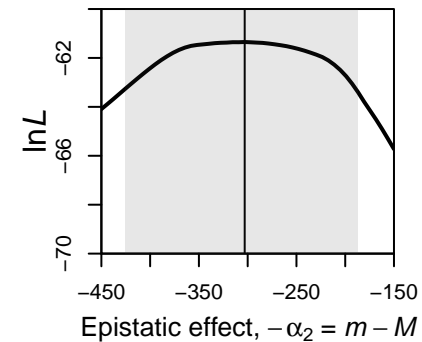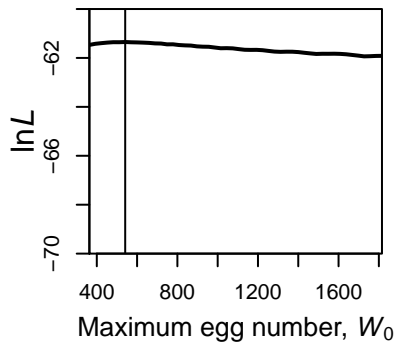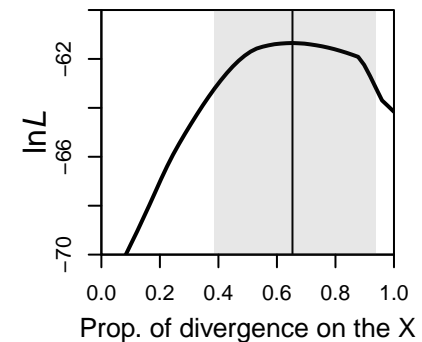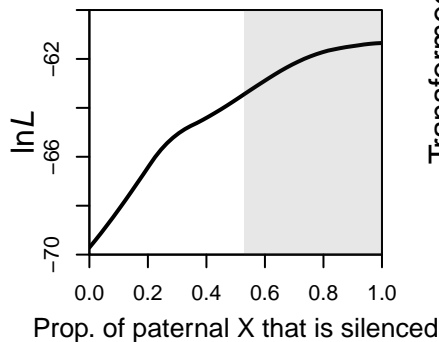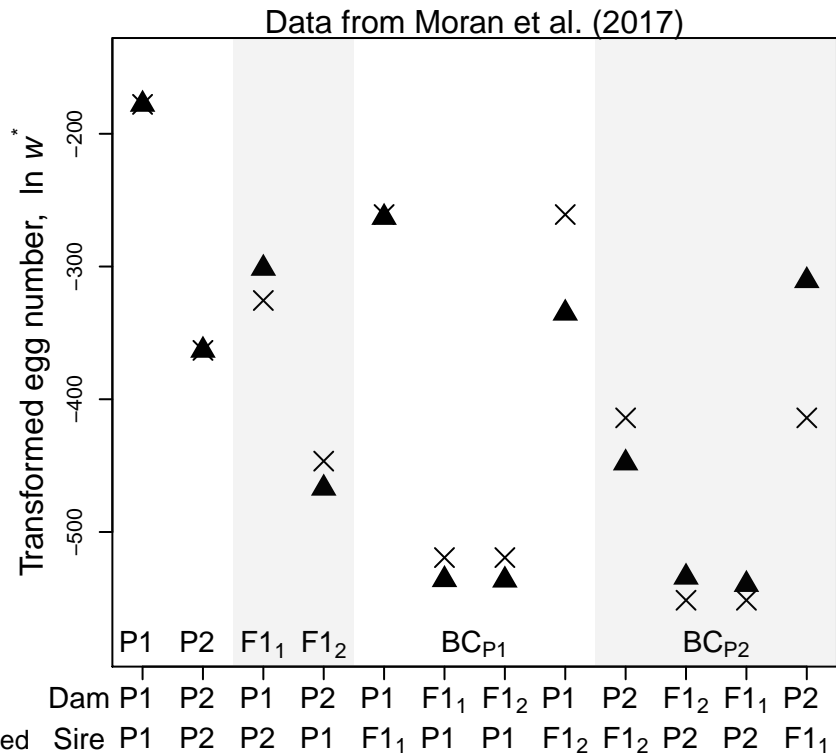

Supplement: Supplementary Figure S3 [file EMS194048-supplement-Supplementary_Figure_S3.pdf]

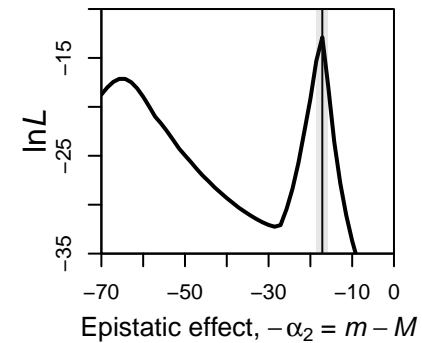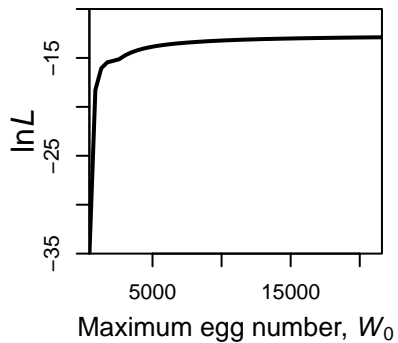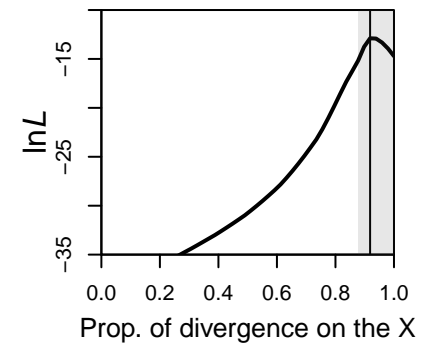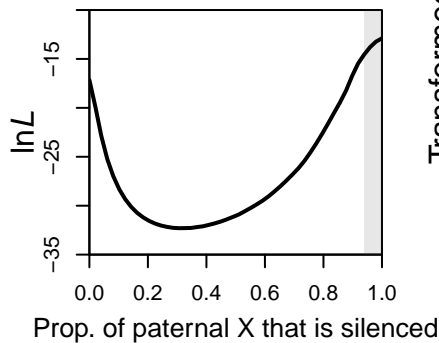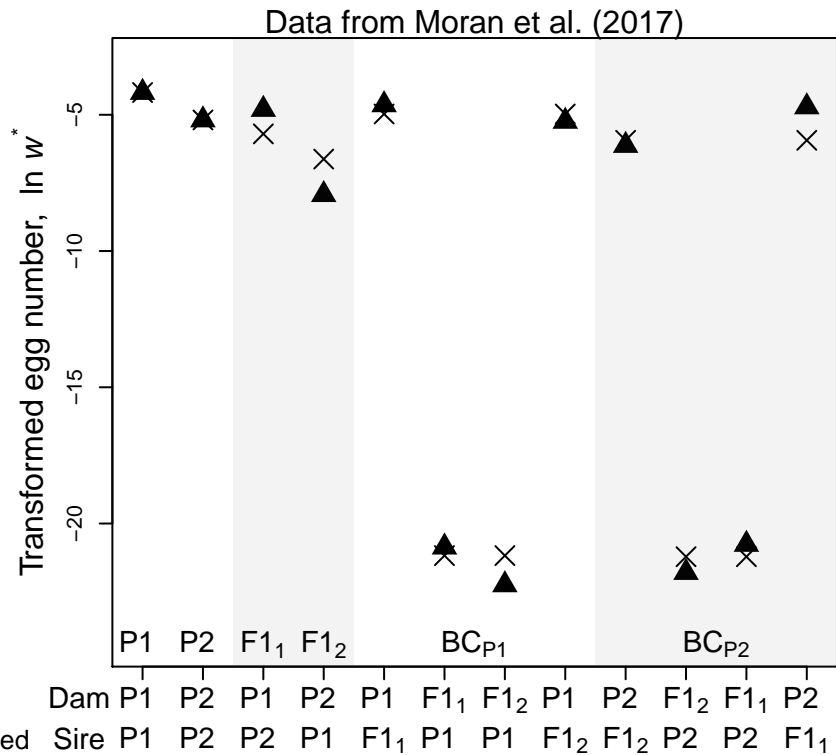

Supplement: Supplementary Figure S4 [file EMS194048-supplement-Supplementary_Figure_S4.pdf]
